# The association of long-term exposure to outdoor air pollution with all-cause GP visits and hospital admissions by ethnicity and country of birth in the United Kingdom

**DOI:** 10.1371/journal.pone.0275414

**Published:** 2023-10-11

**Authors:** Mary Abed Al Ahad

**Affiliations:** School of Geography and Sustainable Development, University of St Andrews, Scotland, United Kingdom; Universal Scientific Education and Research Network, CAMEROON

## Abstract

**Background:**

Air pollution is associated with poor health. Yet, more research is needed to reveal the association of long-term exposure to outdoor air pollution with less studied health outcomes like hospital admissions and general-practitioner (GP) visits and whether this association is stronger for ethnic minorities compared to the rest of population. This study investigates the association between air pollution and all-cause GP visits and hospital admissions by ethnicity in the United-Kingdom (UK).

**Methods:**

We used individual-level longitudinal data from the *“UK Household Longitudinal Study”* including 46,442 adult individuals who provided 140,466 responses across five years (2015–2019). This data was linked to yearly concentrations of NO_2_, SO_2_, and particulate-matter (PM10, PM2.5) outdoor pollution using the Lower Super Output Area (LSOA) of residence for each individual. Multilevel mixed-effects ordered logistic models were used to assess the association between air pollution and all-cause GP visits and hospital admissions.

**Results:**

We found higher odds of hospital admissions per 1 μg/m^3^ increase in annual concentrations of NO_2_ (OR = 1.008; 95%CI = 1.004–1.012), SO_2_ (OR = 1.048; 95%CI = 1.014–1.083), PM10 (OR = 1.011; 95%CI = 1.003–1.018), and PM2.5 (OR = 1.018; 95%CI = 1.007–1.029) pollutants. Higher odds of GP visits were also observed with increased exposure to NO_2_ (OR = 1.010; 95%CI = 1.006–1.014) and SO_2_ (OR = 1.114; 95%CI = 1.077–1.152) pollutants. The observed associations did not differ across ethnic groups, but by country of birth, they were more pronounced in individuals born outside UK than those born in UK.

**Conclusion:**

This study supports an association between higher exposure to outdoor air pollution and increased all-cause hospital admissions and GP visits. Further longitudinal studies with longer follow-up time periods may be able to reveal more definite conclusions on the influence of ethnicity on the association between long-term outdoor air pollution and both hospital admissions and GP visits.

## Introduction

Air pollution is a major threat of human health and planetary wellbeing, which is therefore being addressed by the Conference of The Parities (COP) global initiative whose 26^th^ assembly was held in Glasgow (Scotland) between 31/10/2021-12/11/2021. Effects of short-term outdoor air pollution exposure on human health have been extensively studied over the past two decades [1–5]. Whilst short-term exposure to air pollution is a good indicator for acute health conditions, studying the long-term impact is more appropriate for chronic health problems [6]. This is related to the ability of long-term exposure to capture the cumulative effects of air pollution on health over time, which results in larger estimates compared to short-term exposure [7]. For example, in New England, a 4.2% versus a 0.7% in hospital admissions for all-cause respiratory conditions were noted for every 10 μg/m^3^ increase in long-term versus short-term exposure to particulate matter with diameter ≤ 2.5 μm (PM2.5) pollutant, respectively [8]. Furthermore, the most studied health effect so far is mortality [2, 9], as access to mortality databases requires a less strict ethical approval and death only occurs once in individual`s life.

The association between outdoor air pollution and human health is also influenced by demographic (e.g., age, gender) [2], individual`s lifestyle and baseline health (e.g., pre-existing disease, obesity, cigarette smoking, alcohol drinking, physical exercise) [10–13], socioeconomic (e.g., ethnicity, country of birth, social hierarchisation, level of education, occupation, level of income, neighbourhood, conditions of accommodation, area-level multiple deprivation index, rural-urban classifications, population density) [2, 14–19] and environmental (e.g., season, temperature, relative humidity, altitude, rainfall and wind) [20–22] factors. For example, prevoius studies have shown that older people are more vulnerable to the health impacts of both short- and long-term exposure to air pollution due to the physiological degeneration of the human body with increasing age, lower immunity and antioxidant defence, and the emergence of chronic health conditions [23–25]. Some studies have also shown that the association between short-term exposure to air pollution and health is stronger for females compared to males [10, 26, 27], whereas other studies have shown a stronger association between both short- and long-term exposure to air pollution and health among males [28–30]. Exposure to long-term air pollution was shown to affect more the health of people with low educational levels and higher socioeconomic deprivation compared to those with high educational levels and good socioeconomic position [30–32]. Additionally, exposure to both short- and long-term air pollution has a higher impact on health in summer compared to winter seasons because high temperatures and sunlight facilitate the production of ozone pollution [33, 34], while rainfall reduces air pollution by washing down particulate pollutants [35]. Among all these demographic, socioeconomic and environmental factors, ethnicity is especially important, as it reflects the socioeconomic factors beyond genetic factors [36–40]. However, studies deeply assessing the influence of ethnicity on the health impact of outdoor air pollution have mainly been conducted in USA [41–43], which has a different ethnic composition than UK.

Studies whereby temporal and spatial variations of the association between outdoor air pollution and health outcomes have been assessed are also infrequent in the literature [44, 45]. The Between-Within approach of statistical analysis decomposes the overall effect of air pollution on health into Between (spatial: area-related) and Within (temporal: time-related) effects. In the current study, the Between effect is calculated as the average air pollution concentration across the follow-up time for each geographical area (e.g., census output area), while the Within effect is calculated the yearly deviation in air pollution concentrations from the average concentration for each geographical area [45–47]. In other words, the Between effect presents the area-specific average air pollution exposure across the entire follow-up time whereas the Within effect compares years with high or low air pollution exposures to the average air pollution exposure for each area across the follow-up time. This approach would determine whether living in a more polluted geographical area across the follow-up time is the main reason for poor health outcomes or whether it is the changes in air pollution exposure for each year relative to the follow-up average that is leading to poor health.

Taken collectively, this study utilises longitudinal individual-level data linked to air pollution data at the level of Lower Super Output Areas (LSOAs) to investigate the association between five years exposure to outdoor air pollution and all-cause GP visits and hospital admissions by ethnicity and country of birth in the UK. We specifically aimed (1) to examine the overall and the Between-Within (spatial-temporal) effects of five years exposure to four air pollutants (nitrogen dioxide—NO_2_, sulphur dioxide—SO_2_, and particulate matter with diameters of ≤ 10 μm—PM10, and ≤ 2.5 μm—PM2.5) on all-cause GP visits and hospital admissions; (2) to assess whether the association between air pollution and GP visits and hospital admissions is stronger for ethnic minorities compared to British-white ethnicity; and (3) to investigate whether the association between air pollution and GP visits and hospital admissions is greater for non-UK-born compared to UK-born individuals.

## Methods

This article conforms to the Strengthening the Reporting of Observational Studies in Epidemiology (STROBE) guidelines for reporting an original article [48].

### Study design and population

This study applies a prospective longitudinal panel design and utilises individual-level longitudinal data from the “*Understanding Society*: *The UK Household Longitudinal Study (UKHLS)*” on 46,442 adult individuals (age 16+) who provided a total of 140,466 responses across four data collection waves (waves 7–10) over five years (2015–2019). The UKHLS is a rich dataset, which consists of 10 data collection waves from 2009 to 2020 with around 40,000 households recruited at wave 1 from the four nations of the UK: England, Wales, Scotland, and Northern Ireland. Further information on the design and variables collected by the UKHLS are described elsewhere [46, 49–51]. We only used data from waves 7–10 of the UKHLS adult survey due to the unavailability of data on hospital admissions and GP visits in the earlier waves. We have to also note that the initial adult survey of the UKHLS for waves 7–10 involved a total of 49,028 individuals with 151,834 responses and that 11,368 responses were omitted because of duplicate responses from the same individual within the same year (*n* = 3,429), missing air pollution data for the year 2020 (*n* = 1,046), missing the year variable (*n* = 14), missing the LSOA or local authority of residence (*n* = 71), missing age (*n* = 41), and missing hospital admissions or GP visits responses (*n* = 6,767).

### Air pollution data and its linkage to the UKHLS individual-level data

We obtained yearly outdoor air pollution data that combine all sources of air pollution including road traffic and industrial/combustion processes from the “Department for Environment Food and Rural Affairs (DEFRA)” database [52]. The DEFRA database includes yearly outdoor modelled air pollution data from 2001 to 2019 for several pollutants including NO_2_, SO_2_, PM10, PM2.5, NO_x_ (nitrogen oxides), ozone, and benzene. These are raster data of mean annual concentrations of pollutants measured in μg/m^3^, estimated using air dispersion modelling (Pollution Climate Mapping) at a spatial resolution of 1x1 km^2^ and projected using the UK National Grid [53]. The DEFRA data also include population-weighted annual mean concentrations of PM2.5 for each Local Authority starting the year of 2010. Detailed information on this dataset is available at the DEFRA website [52].

For this study, we chose four pollutants (NO_2_, SO_2_, PM10, and PM2.5) from the DEFRA database. This is because these pollutants have been shown by relevant literature to be related to poor health outcomes. We did not study ozone because this is a seasonal pollutant that interacts with other pollutants in the atmosphere, mostly during the summer [2, 30, 39]. Thus, annual pollution data is not suitable to study the health effects of ozone.

The annual 1x1 km^2^ raster air pollution data for each of NO_2_, SO_2_, PM10, PM2.5 pollutants were linked to the UKHLS individual-level data using the Lower Super Output Areas (LSOAs; data zones for Scotland and Super Output Areas for Northern Ireland) of residence for each individual in each year (2015–2019). LSOAs are used to decompose England and Wales based on the population size into areas with around 1000 to 3000 individuals and are the lowest level of geography offered by the UKHLS dataset. Some LSOAs, especially those located in urbanised areas, have an area of less than 1x1 km^2^, which offers high spatial resolution to assess the impact of air pollution on hospital admissions and GP visits. The LSOAs in England and Wales are equivalent to data zones in Scotland and to Super Output Areas in Northern Ireland. For simplicity we refer to the joint LSOAs, data zones, and Super Output Areas as LSOAs.

The linkage was done by calculating an area-weighted average air pollution concentration of each of the four pollutants for each LSOA in each year based on the proportion of area intersection between the 1x1 km^2^ raster squares and the respective LSOA. For example, if a LSOA intersected with three 1x1 km^2^ squares in which one intersection covered half of the area of that LSOA while the other two intersections covered a proportion of 0.3 and 0.2, respectively; the air pollutant concentration for that LSOA would be 0.5×air pollutant concentration of the first intersected square + 0.3×air pollutant concentration of the second intersected square + 0.2×air pollutant concentration of the third intersected square.

After this process, each LSOA in the UK had an air pollution concentration for each of the four pollutants (NO_2_, SO_2_, PM10, PM2.5) for each year between 2015 and 2019, which we referred to as the overall pollutant effect. We also calculated the five years (2015–2019) average concentration of each air pollutant for each LSOA, this was referred to as the *Between* pollutant effect, which reveals spatial associations. Then, we calculated the yearly deviation in the air pollutants concentrations from the five years mean for each LSOA in each year, which was referred to as the *Within* pollutant effect. This *Within* effect represents the annual temporal trend of air pollution across the five years of follow-up within each LSOA in the present study. Finally, the LSOA of residence for each individual in the UKHLS data was used to link the overall, Between (spatial), and Within (temporal) concentrations of the four pollutants for each year between 2015 and 2019.

### Variables and definitions

#### Outcome variables

This study included two ordered categorical outcomes from the UKHLS dataset. These are GP visits and outpatient hospital admissions, which were collected by asking individuals on the number of times they visited a GP (i.e., a general-practitioner; any type of doctor including general, family, or specialty doctor) or went to a hospital/clinic as outpatients in the past 12 months preceding the data collection date as follows: 0 = none, 1 = one to two times, 2 = three to five times, 3 = six to ten times, and 4 = more than ten times. The question did not ask either about the exact number of GP visits and hospital admissions or about the specific reason behind the GP or hospital admission visit, but rather referred to all-causes visits. Thus, a visit to a GP could be for a disease complication or for a simple routine consultation, and a visit to a hospital could be for any reason that does not require an overnight stay at the hospital (i.e., only outpatient hospital admissions). Inpatient hospital admissions for overnight stays are not included in this study.

#### Air pollution independent variables

This study included four air pollution independent variables: NO_2,_ SO_2,_ PM10, and PM2.5 pollutants. These are continuous variables measured as the mean annual concentrations of outdoor air pollutants in μg/m^3^ at the LSOA of residence for each individual in each year. We also included the *Between* (spatial) and *Within* (temporal) variables for each of the four pollutants.

#### Socioeconomic and lifestyle covariates

For this study, we selected a number of individual-level demographic, socioeconomic, and lifestyle covariates based on what is available in the UKHLS dataset and based on the confounders and effect modifiers considered by relevant literature [2, 9]. These included gender (1 = male; 2 = female); age (coded as 16–18 and then in 5 years increments as 19–23; 24–28; 29–33; 34–38; 39–43; 44–48; 49–53; 54–58; 59–63; 59–63; 64–68; 69–73; 74–78; >78); ethnicity (1 = British-white; 2 = Other-white; 3 = Indian; 4 = Pakistani/Bangladeshi; 5 = Black/African/Caribbean; 6 = mixed ethnicities; 7 = Other ethnicities); country of birth (1 = born in UK; 2 = not born in UK; 3 = No answer); marital status (1 = married; 2 = living as a couple; 3 = widowed; 4 = divorced/separated; 5 = single never married; 6 = no answer); education (1 = university degree; 2 = high school degree; 3 = lower educational levels; 4 = other qualifications; 5 = still a student); perceived financial situation (1 = living comfortably/doing alright; 2 = living difficultly; 3 = no answer); socioeconomic classification (1 = management and professionals occupations; 2 = intermediate occupations; 3 = routine occupations; 4 = not applicable: Student/retired/Not working; 5 = no answer); smoking status (0 = non-smoker; 1 = smoker; 2 = no answer); and rural-urban indicator (1 = urban; 2 = rural). It should be noted that for the ethnicity variable, Indian, Pakistani/Bangladeshi, Black/African/Caribbean, mixed ethnicities and other ethnic groups are considered to be ethnic minorities in the context of the UK [54].

Finally, year dummies (calendar year: 2015–2019) were considered as a control for the time trend in our analysis following the approach of relevant studies [55, 56]. Given that our study utilises yearly air pollution data, controlling for other temporal covariates considered by relevant literature such as seasonal trends [26, 57] was not possible.

### Statistical analysis

Percentages were computed to describe the ordered categories of the number of GP visits and hospital admissions, and the categories of the individuals’ socioeconomic and lifestyle covariates for each wave (waves 7–10) of the UKHLS sample. We also examined the correlation between the overall concentrations of NO_2_, SO_2_, PM10, and PM2.5 pollutants across the five years of follow-up using Pearson’s correlation coefficient. Given the high observed correlations between the pollutants (Pearson’s coefficient ≥ 0.7 [58]; Table 2) which could be due to the emission sources of the pollutants and the atmospheric reactions between them, the association of NO_2_, SO_2_, PM10, and PM2.5 pollutants with GP visits and hospital admissions was examined in separate regression models. In other words, each of the four pollutants was included in a separate model, rather than including all the four pollutants in one model.

To account for the study design which involves linkages of air pollution to individual-level data at the LSOAs level and to account for the repeated individuals’ responses across time, the association of the GP visits and outpatient hospital admission ordered outcomes with each of NO_2_, SO_2_, PM10, and PM2.5 pollutants was assessed using three-levels (repeated individual responses across time nested within LSOAs) mixed-effects ordered logistic models [59–61]. These models were adjusted for year (2015–2019) dummies and for the demographic, socioeconomic, lifestyle, and contextual covariates in a stepwise manner. Model 1 was adjusted for age, gender, and year dummies. Model 2 additionally included ethnicity and country of birth. Model 3 accounted for all mentioned variables plus marital status, education, perceived financial situation, and socioeconomic classification. Model 4 adjusted in addition for smoking status and model 5 for rural-urban indicator (S1 Table in S1 File). Akaike Information Criterion (AIC) and Bayesian information criterion (BIC) were used to determine goodness of fit of models with a smaller AIC or BIC indicating better fit modelling. The results of Models 1 to 5 with their respective AIC and BIC are found in S2 Table in S1 File. In the main manuscript, we show only models 4 and 5. The structuring of model 5 is presented in Eq (1). In a supplementary analysis, we also demonstrate the association of GP visits and outpatient hospital admissions with each of the socioeconomic and lifestyle confounders in model 5 (S3 and S4 Tables in S1 File).

$$\log(\frac{{Y_{\mathrm{tij}}}^{\left(s\right)}}{1-{Y_{\mathrm{tij}}}^{\left(s\right)}})={\beta_{0}}^{\left(s\right)}+U_{0ij}+U_{0j}+{\beta_{1}\;overall\;pollutant\;concentration}_{tij}+{\beta_{2}\;Age}_{tij}+{\beta_{3}\;Gender}_{tij}+{\beta_{4}\;Ethnicity}_{tij}+{\beta_{5}\;Country\;of\;birth}_{tij}+{\beta_{6}\;Marital\;status}_{tij}+{\beta_{7}\;Education}_{tij}+{\beta_{8}\;Socioeconomic\;classification}_{tij}+{\beta_{9}\;Perceivedfinancial\;situation}_{tij}+{\beta_{10}\;Smoking\;status}_{tij}+{\beta_{11}\;Year\;dummies}_{ij}+{\beta_{12}\;Rural\;urban\;indicator}_{tij}+\varepsilon_{tij}$$ (1)

In further analysis, the multilevel mixed-effects ordered logistic models were used to examine the *Between* (spatial) and *Within* (temporal) effects (Eq (2)) of air pollution on GP visits and outpatient hospital admissions.

$$\log(\frac{{Y_{\mathrm{tij}}}^{\left(s\right)}}{1-{Y_{\mathrm{tij}}}^{\left(s\right)}})\;=\;{\beta_{0}}^{\left(s\right)}+U_{0ij}+U_{0j}+\;{\beta_{1}\;Between\;pollutant\;concentration}_{tij}+\;{\beta_{2}\;Within\;pollutant\;concentration}_{tij}+\;{\beta_{3}\;Age}_{tij}+\;{\beta_{4}\;Gender}_{tij}+\;{\beta_{5}\;Ethnicity}_{tij}+{\beta_{6}\;Country\;of\;birth}_{tij}+\;{\beta_{7}\;Marital\;status}_{tij}+\;{\beta_{8}\;Education}_{tij}+\;{\beta_{9}\;Socioeconomic\;classification}_{tij}+{\beta_{10}\;Perceived\;financial\;situation}_{tij}+\;{\beta_{11}\;Smoking\;status}_{tij}+\;{\beta_{12}\;Year\;dummies}_{ij}+{\beta_{13}\;Rural\;urban\;indicator}_{tij}+\varepsilon_{tij}\;$$ (2)

where *Y*_*tij*_ is the outcome (GP visits or outpatient hospital admissions) for individual *i*, in LSOA *j* at year *t*; *s* is the level of an ordered category with s levels for GP visits or hospital admission outcomes; *β*_1_, *β*_2_ …. *β*_13_ are the slopes of fixed effects; *β*_0_ is the fixed intercept; *ε*_*tij*_ are the model residuals; *U*_0*ij*_ is level 2 random intercept of individuals nested in LSOAs; *U*_0*j*_ is level 3 random intercept of LSOAs; the conditional distribution of the response given the random effects is assumed to be multinomial, with success probability determined by the logistic cumulative distribution function; *Between* pollutant concentrations represent the average air pollutant concentration for each LSOA across the five years of follow-up–thus assessing the spatial LSOA-related effects of air pollution; *Within* pollutant concentrations represent the yearly difference of the air pollutant concentration from the LSOA five years average–thus assessing the temporal-related effects of air pollution within each LSOA; the pollutant concentration is for NO_2,_ SO_2,_ PM10, or PM2.5 where each pollutant is entered in a separate model; the four pollutants were not included in the same model due to high correlation as noted before.

Bonferroni [62] and Šidák [63] familywise error rates were used to obtain corrected multiple comparisons critical value for the P-values in models 4 and 5 of the four pollutant models. This was done because each of the four air pollutants was run in a separate model. In other words, we had one model for NO_2,_ another model for SO_2,_ another model for PM10, and another model for PM2.5. This means that in each of models 4 and 5 for each of the GP visits and outpatient hospital admissions outcomes, we had a set of four P-values where each P-value is estimated from each of the NO_2,_ SO_2,_ PM10, and PM2.5 separate models.

In a sensitivity analysis, we also accounted for spatial autocorrelation in air pollution concentrations for neighbouring LSOAs by adjusting the models for the local spatial autocorrelation Getis-Ord Gi* statistical measure [64, 65]. Accounting for spatial autocorrelation in spatial-related measurements such as air pollution is important to reduce type I errors in derived estimates [66]. The Getis-Ord Gi* measure was calculated for each of the four pollutants (NO_2,_ SO_2,_ PM10, and PM2.5) in each year between 2015 and 2019 for each LSOA by constructing a Fixed Distance Band spatial weight matrix using a cutoff radius distance of 5 Km, which ensured that every LSOA has at least one neighbouring LSOA within the 5 Km radius around its centroid. The Fixed Distance Band method was chosen in determining what forms a neighbourhood for each LSOA over other methods (e.g., K-nearest neighbour or Neighbour connectivity) as it ensured that all LSOAs whether small or large in size are treated using a similar criterion. Given that LSOAs are constructed based on population size rather than area size, using the Fixed Distance Band method with a cutoff value of 5 Km ensures that only LSOAs whose centroids fall within the 5 Km radius of a specific LSOA centroid are treated as neighbours for that specific LSOA no matter of the area size of that LSOA. Further information on the calculation of Getis-Ord Gi* measure and its formula can be found in the ArcGIS Pro documentation [67]. In summary, Getis-Ord Gi* measure determines which LSOAs are spatially correlated with high air pollutant concentrations (hot spots) or low air pollutant concentrations (cold spots) at a 99%, 95%, and 90% confidence levels or not significantly spatially correlated by relying on z-scores and P-values. S2 File includes maps showing the Getis-Ord Gi* hot-cold spot analysis for each of NO_2,_ SO_2,_ PM10, and PM2.5 pollutants across the LSOAs in the UK for each year between 2015 and 2019.

Finally, we incorporated into the multilevel ordered logistic models an interaction term between ethnicity and each of NO_2_, SO_2_, PM10, and PM2.5 pollutants and between country of birth and each of the four pollutants to assess whether the association between air pollution and GP visits and hospital admissions varies between ethnic groups and by country of birth. Interaction terms were incorporated into the overall pollutant models and into the *Between-Within* models, each at a time. Ethnicity was assessed separately from country of birth.

Statistical analysis was performed in STATA software (StataCorp. 2015. Stata Statistical Software: Release 14. College Station, TX: StataCorp LP) and spatial pre-processing was conducted using ArcGIS Pro software. Regression results were reported in terms of odds ratios (ORs) and 95% confidence intervals (CIs) for the increase in the ordered categories of the number of GP visits and hospital admissions per 1 μg/m^3^ increase in the air pollutants. Statistical significance was considered at a P-value of less than 0.05.

### Ethical considerations

This paper was granted ethical approval on the 14^th^ of May 2020 by the author’s affiliated institution (School of Geography and Sustainable Development Ethics Committee, acting on behalf of the University Teaching and Research Ethics Committee (UTREC) at the University of St Andrews). The paper uses secondary adult (age 16+) pseudo-anonymised data from the “Understanding Society: The UK Household Longitudinal Study (UKHLS)” and authors did not have access to potentially identifying information; thus, obtaining participants’ informed consent is not applicable and was waved by the authors’ institution ethics committee. The University of Essex responsible for the UKHLS data collection and management has already obtained written informed consent from all the study participants and ensured adherence to the Helsinki Declaration [49].

## Results

### Description of GP visits, hospital admissions and individuals’ socioeconomic and lifestyle covariates

In all the four waves, most of the individuals visited a GP one to two times, did not go to a hospital as outpatients, were females, aged between 34 and 58 years old, had British-white ethnicity, were born in the UK, were married, had a university degree or other educational qualifications, reported a comfortable/alright financial situation, had a managerial or professional socioeconomic classification (if working), were non-smokers, resided in an urban area, and lived in England (Table 1).

**Table 1:** Description of GP visits, outpatient hospital admissions and individual’s socioeconomic and lifestyle covariates for each wave of the UKHLS sample (*N* = 140,466 surveys from 46,442 individuals).

		Wave7 (2015–2017)	Wave8 (2016–2018)	Wave9 (2017–2019)	Wave10 (2018–2019)
		*N* = 38,663	*N* = 36,319	*N* = 33,727	*N* = 31,757
		Percent	Percent	Percent	Percent
**GP visits**	None	26.7	21.1	21.2	28.3
	One to two times	37.0	39.5	40.8	40.6
	Three to five times	20.8	23.1	23.2	19.7
	Six to ten times	8.5	9.3	9.1	6.8
	More than ten times	7.0	6.9	5.7	4.7
**Outpatient hospital admissions**	None	57.0	54.3	53.0	55.5
	One to two times	25.1	27.3	28.0	27.4
	Three to five times	10.1	10.7	11.5	10.3
	Six to ten times	4.3	4.4	4.4	3.9
	More than ten times	3.6	3.3	3.2	2.9
**Gender**	Male	44.5	44.7	44.3	44.3
	Female	55.5	55.3	55.7	55.7
**Age**	Young (<34)	24.6	23.8	23.1	22.5
	Middle age (34–58)	43.9	43.3	42.8	42.3
	Old (>58)	31.5	32.9	34.1	35.3
**Ethnicity**	British-white	74.5	75.0	76.3	77.3
	Other-white	5.7	5.6	5.4	5.2
	Indian	4.2	4.2	3.9	3.8
	Pakistani/Bangladeshi	5.6	5.7	5.7	5.5
	Black/African/Caribbean	4.8	4.5	4.0	3.6
	Mixed ethnicities	1.8	1.9	1.8	1.8
	Other ethnicities	3.3	3.3	2.9	2.8
**Country of birth**	Born in the UK	65.6	66.4	67.3	68.1
	Not born in the UK	15.9	15.1	14.0	13.0
	No answer	18.5	18.5	18.7	18.9
**Marital status**	Married	52.4	52.9	53.4	53.8
	Living as a couple	10.5	10.2	9.8	9.6
	Widowed	6.0	6.0	6.1	6.0
	Divorced/separated	7.9	7.7	7.9	7.9
	Single never married	23.1	23.1	22.6	22.3
	No answer	0.2	0.2	0.3	0.4
**Education**	University degree	30.1	31.0	31.9	33.1
	High school degree	25.9	26.4	26.7	26.4
	Lower educational levels	1.0	1.0	1.0	1.0
	Other qualifications	36.3	35.4	34.3	33.7
	Still a student	6.7	6.2	6.0	5.8
**Perceived financial situation**	living comfortably/doing alright	71.7	72.4	70.7	71.1
	living difficultly	28.1	27.3	28.9	28.5
	No answer	0.2	0.3	0.4	0.4
**Socio-economic classification**	Management and professional occupations	24.0	23.8	23.5	23.2
	Intermediate occupations	13.3	13.3	12.9	12.6
	Routine occupations	19.7	18.9	18.3	17.4
	NA: student/retired/not working	42.4	43.0	43.1	43.9
	No answer	0.6	1.0	2.2	2.9
**Smoking status**	Non-smoker	84.4	85.2	86.5	86.9
	Smoker	15.5	14.7	13.4	12.9
	No answer	0.1	0.2	0.1	0.2
**Rural-urban indicator**	Urban	76.7	76.3	75.6	75.3
	Rural	23.3	23.7	24.4	24.7
**Nation**	England	79.0	78.9	78.5	78.4
	Wales	6.4	6.4	6.5	6.5
	Scotland	8.3	8.3	8.4	8.5
	Northern Ireland	6.3	6.4	6.6	6.7

## Description of air pollution

**Fig 1:**
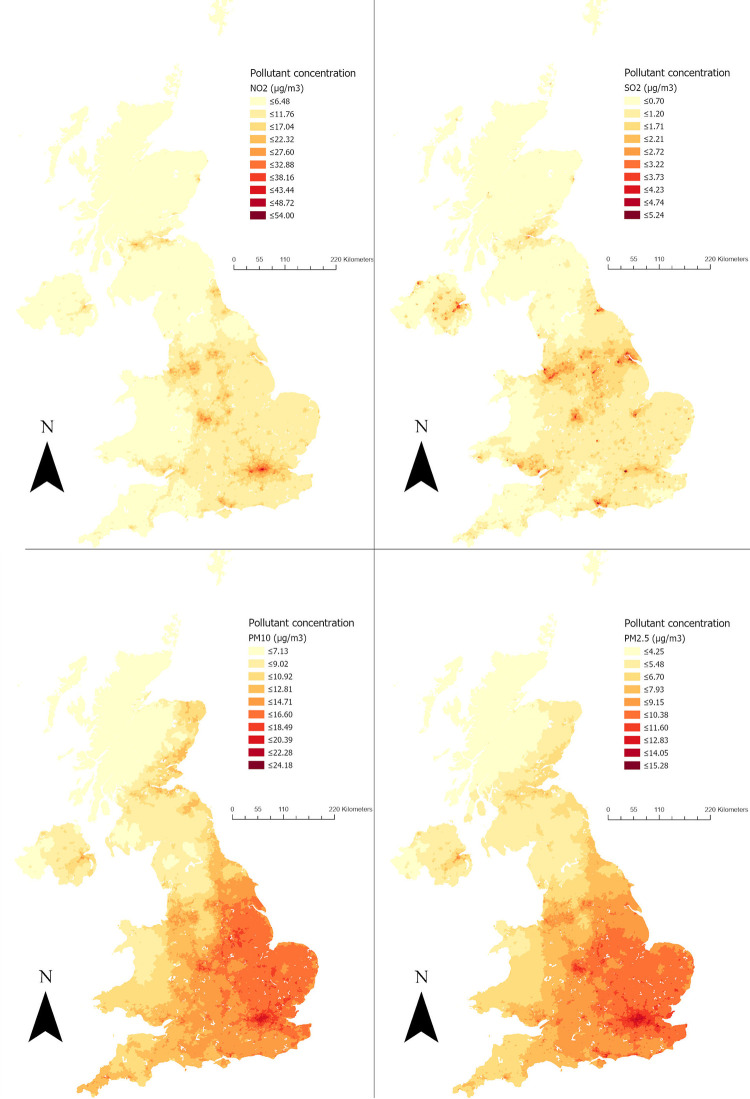
Four maps showing the average yearly concentrations of NO_2_, SO_2_, PM10, and PM2.5 pollutants across the five years of follow-up (2015–2019) for each LSOA in the UK. The maps were constructed by the author in ArcGIS Pro software using air pollutants shapefiles between 2015 and 2019 from the DEFRA data repository [52] and LSOAs and data zones 2011 UK boundaries from the Office for National Statistics, National Records of Scotland, and Northern Ireland Statistics [68]. Both DEFRA and Office for National Statistics shapefiles are governed under the Open Government Licence v.3.0.

A high correlation (Pearson’s coefficient ≥ 0.7) was observed between NO_2_, PM10, and PM2.5 pollutants, which could be explained by the chemical reactions between particulate matter and NO_2_ pollutants in the atmosphere. Across the five years of follow-up (2015–2019), the mean of NO_2_ was 14.05 μg/m^3^ (SD = 6.67), the mean of SO_2_ was 1.45 μg/m^3^ (SD = 0.61), the mean of PM10 was 13.41 μg/m^3^ (SD = 3.05), and the mean of PM2.5 was 8.74 μg/m^3^ (SD = 2.14) (Table 2). The average yearly concentrations of NO_2,_ SO_2,_ PM10, and PM2.5 pollutants across the five years of follow-up (2015–2019) for each LSOA in the UK are also demonstrated in Fig 1. As expected, major urban areas such as London, south and middle of England, and the central belt of Scotland are the most polluted. The average yearly variations in air pollutants from the five years mean for each year between 2015 and 2019 are shown in S5 Table in S1 File, in which NO_2_ showed a relatively high difference in 2016 from the five years average NO_2_ (mean difference = 1.58 from the five years average of 14.08).

**Table 2:** Exposure description and correlation matrix of air pollutants (*N* = 42,619 LSOAs; time = 2015–2019).

	Pearson’s correlation coefficient							
	NO_2_ (μg/m^3^)	SO_2_ (μg/m^3^)	PM10 (μg/m^3^)	PM2.5 (μg/m^3^)	Mean	SD	Median	Interquartile range
NO_2_ (μg/m^3^)	1.00				14.08	6.67	13.28	8.40
SO_2_ (μg/m^3^)	0.54	1.00			1.45	0.61	1.35	0.76
PM10 (μg/m^3^)	**0.72**	0.42	1.00		13.41	3.05	13.59	4.55
PM2.5 (μg/m^3^)	**0.74**	0.46	**0.98**	1.00	8.74	2.14	8.91	3.26

Strong correlations with correlation coefficient ≥0.70 are highlighted in bold.

## The association of air pollution with all-cause GP visits and hospital admissions

Results showed that higher visits to a GP are associated with higher concentrations of NO_2_ (OR = 1.010, 95%CI = 1.006–1.014) and SO_2_ (OR = 1.114, 95%CI = 1.077–1.152) pollutants (Table 3). Higher odds of all-cause outpatient hospital admissions were also observed with increased concentrations of NO_2_ (OR = 1.008, 95%CI = 1.004–1.012), SO_2_ (OR = 1.048, 95%CI = 1.014–1.083), PM10 (OR = 1.011, 95%CI = 1.003–1.018) and PM2.5 (OR = 1.018, 95%CI = 1.007–1.029) pollutants (Table 4). Adjusting for spatial autocorrelation using Getis-Ord Gi* measurement showed similar results, however, increasing concentrations of PM10 and PM2.5 pollutants are now showing an association with higher odds of GP visits and increased exposure to SO_2_ is not significantly associated anymore with higher odds of outpatient hospital admissions (S6 Table in S1 File).

**Table 3:** The association of air pollution with all-cause GP visits (N = 140,466 surveys from 46,442 individuals).

	Model 4	Model 5
	OR [95%CI]	OR [95%CI]
**Overall pollutant effect**		
NO_2_ (μg/m^3^)	1.011 [1.008, 1.015]**^ǂ^ ^¥^	1.010 [1.006, 1.014]**^ǂ^ ^¥^
SO_2_ (μg/m^3^)	1.127 [1.091, 1.163]**^ǂ^ ^¥^	1.114 [1.077, 1.152]**^ǂ^ ^¥^
PM10 (μg/m^3^)	1.003 [0.996, 1.011]	0.999 [0.992, 1.007]
PM2.5 (μg/m^3^)	1.006 [0.995, 1.017]	0.999 [0.987, 1.010]
**Between pollutant effect**		
NO_2_ (μg/m^3^)	1.011 [1.008, 1.015]**^ǂ^ ^¥^	1.010 [1.005, 1.014]**^ǂ^ ^¥^
SO_2_ (μg/m^3^)	1.134 [1.088, 1.18]**^ǂ^ ^¥^	1.115 [1.067, 1.166]**^ǂ^ ^¥^
PM10 (μg/m^3^)	1.006 [0.998, 1.015]	1.001 [0.993, 1.010]
PM2.5 (μg/m^3^)	1.012 [0.999, 1.024]	1.003 [0.990, 1.016]
**Within pollutant effect**		
NO_2_ (μg/m^3^)	1.001 [0.978, 1.023]	1.000 [0.977, 1.022]
SO_2_ (μg/m^3^)	1.052 [0.959, 1.154]	1.057 [0.963, 1.158]
PM10 (μg/m^3^)	1.005 [0.979, 1.032]	1.004 [0.977, 1.031]
PM2.5 (μg/m^3^)	0.997 [0.961, 1.035]	0.994 [0.958, 1.032]

**P-value <0.01

*P-value<0.05

^ǂ^P-value < Bonferroni [62] familywise error rate corrected critical value of 0.0127 for four multiple pollutant comparisons for each of models 4 and 5 and each of the overall, Between, and Within pollutant effects

^¥^P-value < Šidák [63] familywise error rate corrected critical value of 0.0125 for four multiple pollutant comparisons for each of models 4 and 5 and each of the overall, Between, and Within pollutant effects.

ORs and 95%CIs are expressed in terms of 1 μg/m^3^ increase in the air pollutants; Model 4 is adjusted for age, gender, ethnicity, country of birth, marital status, education, perceived financial situation, socioeconomic classification, smoking status, and year dummies (2015–2019); Model 5 is additionally adjusted for rural-urban indicator.

**Table 4:** The association of air pollution with all-cause outpatient hospital admissions (*N* = 140,466 surveys from 46,442 individuals).

	Model 4	Model 5
	OR [95%CI]	OR [95%CI]
**Overall pollutant effect**		
NO_2_ (μg/m^3^)	1.010 [1.006, 1.013]**^ǂ^ ^¥^	1.008 [1.004, 1.012]**^ǂ^ ^¥^
SO_2_ (μg/m^3^)	1.067 [1.035, 1.101]**^ǂ^ ^¥^	1.048 [1.014, 1.083]**^ǂ^ ^¥^
PM10 (μg/m^3^)	1.014 [1.007, 1.022]**^ǂ^ ^¥^	1.011 [1.003, 1.018]**^ǂ^ ^¥^
PM2.5 (μg/m^3^)	1.023 [1.013, 1.034]**^ǂ^ ^¥^	1.018 [1.007, 1.029]**^ǂ^ ^¥^
**Between pollutant effect**		
NO_2_ (μg/m^3^)	1.011 [1.007, 1.014]**^ǂ^ ^¥^	1.009 [1.005, 1.013]**^ǂ^ ^¥^
SO_2_ (μg/m^3^)	1.056 [1.016, 1.098]**^ǂ^ ^¥^	1.026 [0.984, 1.070]
PM10 (μg/m^3^)	1.016 [1.008, 1.024]**^ǂ^ ^¥^	1.012 [1.004, 1.020]**^ǂ^ ^¥^
PM2.5 (μg/m^3^)	1.026 [1.015, 1.038]**^ǂ^ ^¥^	1.020 [1.007, 1.032]**^ǂ^ ^¥^
**Within pollutant effect**		
NO_2_ (μg/m^3^)	0.995 [0.971, 1.019]	0.993 [0.970, 1.017]
SO_2_ (μg/m^3^)	1.013 [0.918, 1.118]	1.021 [0.925, 1.127]
PM10 (μg/m^3^)	1.004 [0.976, 1.033]	1.003 [0.975, 1.031]
PM2.5 (μg/m^3^)	0.989 [0.951, 1.029]	0.986 [0.948, 1.026]

**P-value <0.01

*P-value<0.05

^ǂ^P-value < Bonferroni [62] familywise error rate corrected critical value of 0.0127 for four multiple pollutant comparisons for each of models 4 and 5 and each of the overall, Between, and Within pollutant effects

^¥^P-value < Šidák [63] familywise error rate corrected critical value of 0.0125 for four multiple pollutant comparisons for each of models 4 and 5 and each of the overall, Between, and Within pollutant effects.

ORs and 95%CIs are expressed in terms of 1 μg/m^3^ increase in the air pollutants; Model 4 is adjusted for age, gender, ethnicity, country of birth, marital status, education, perceived financial situation, socioeconomic classification, smoking status, and year dummies (2015–2019); Model 5 is additionally adjusted for rural-urban indicator.

Decomposing the overall effect of air pollution on GP visits and hospital admissions into *Between* (spatial: across LSOAs) and *Within* (temporal: across years within each LSOA) effects, showed significant positive associations for the *Between* effect on GP visits for NO_2_ (OR = 1.010, 95%CI = 1.005–1.014) and SO_2_ (OR = 1.115, 95%CI = 1.067, 1.166) pollutants (Table 3), and a significant *Between* effect on outpatient hospital admissions for all the pollutants, except SO_2_ (Table 4). The *Between* effect analysis confirms to the analysis with adjustment for spatial autocorrelation in S6 Table in S1 File, since in both types of analysis SO_2_ is not showing a significant positive association with outpatient hospital admissions. No significant *Within* effects were noted for all the four pollutants neither on GP visits nor on hospital admissions (Tables 3 and 4).

## The association of air pollution with all-cause GP visits and outpatient hospital admissions by ethnicity and country of birth

Analysis showed no differences between ethnic minorities and British-white for the association of air pollution with all-cause GP visits and outpatient hospital admissions (Tables 5 and 6). Thus, ethnic minorities in the UK do not seem to experience greater health-related effects from exposure to air pollution compared to the rest of population. The only exception was black/African/Caribbean individuals who showed higher odds of outpatient hospital admissions with increasing concentrations of PM10 and PM2.5 pollutants compared to British-white (Table 6). On the contrary, other-white ethnicities showed lower odds of GP visits with increasing concentrations of NO_2,_ PM10, and PM2.5 pollutants compared to British-white (Table 5).

**Table 5:** The overall and *Between-Within* effects of air pollution on all-cause GP visits by ethnicity and country of birth (*N* = 140,466 surveys from 46,442 individuals).

	**Overall pollutant effect**			
**Ethnicity-air pollution interaction models**	NO_2_ (μg/m^3^)	SO_2_ (μg/m^3^)	PM10 (μg/m^3^)	PM2.5 (μg/m^3^)
	OR [95%CI]	OR [95%CI]	OR [95%CI]	OR [95%CI]
British-white (Reference)				
Other-white	0.987 [0.976, 0.998]*	1.034 [0.931, 1.149]	0.972 [0.949, 0.996]*	0.955 [0.922, 0.988]**
Indian	0.979 [0.961, 0.998]*	1.048 [0.888, 1.238]	0.984 [0.944, 1.025]	0.984 [0.924, 1.048]
Pakistani/Bangladeshi	1.003 [0.989, 1.017]	0.992 [0.853, 1.155]	0.982 [0.953, 1.011]	0.986 [0.939, 1.034]
Black/African/Caribbean	1 [0.984, 1.017]	1.085 [0.915, 1.286]	1.021 [0.982, 1.063]	1.031 [0.969, 1.096]
Mixed ethnicities	0.988 [0.968, 1.008]	0.919 [0.727, 1.161]	0.974 [0.927, 1.023]	0.969 [0.899, 1.043]
Other ethnicities	1.003 [0.989, 1.017]	1.044 [0.888, 1.227]	1.042 [1.007, 1.078]*	1.069 [1.017, 1.123]**
**Country of birth-air pollution interaction models**				
Born in UK (Reference)				
Not born in UK	1.019 [1.011, 1.027]**	1.145 [1.046, 1.252]**	1.038 [1.018, 1.058]**	1.062 [1.032, 1.093]**
No answer	1.007 [0.999, 1.017]	1.021 [0.952, 1.095]	1.002 [0.984, 1.020]	0.996 [0.971, 1.021]
	**Between pollutant effect**			
**Ethnicity-air pollution interaction models**	NO_2_ (μg/m^3^)	SO_2_ (μg/m^3^)	PM10 (μg/m^3^)	PM2.5 (μg/m^3^)
	OR [95%CI]	OR [95%CI]	OR [95%CI]	OR [95%CI]
British-white (Reference)				
Other-white	0.986 [0.975, 0.997]*	1.018 [0.893, 1.16]	0.975 [0.95, 1.001]	0.962 [0.927, 0.999]*
Indian	0.99 [0.97, 1.01]	1.063 [0.856, 1.321]	0.997 [0.949, 1.047]	0.998 [0.925, 1.076]
Pakistani/Bangladeshi	1.002 [0.987, 1.016]	1.257 [1.005, 1.573]*	1.015 [0.981, 1.05]	1.017 [0.962, 1.075]
Black/African/Caribbean	1.01 [0.993, 1.028]	1.108 [0.874, 1.405]	1.023 [0.979, 1.07]	1.036 [0.965, 1.113]
Mixed ethnicities	0.992 [0.971, 1.013]	0.92 [0.68, 1.245]	0.985 [0.933, 1.04]	0.975 [0.897, 1.059]
Other ethnicities	1.006 [0.991, 1.021]	1.049 [0.859, 1.28]	1.051 [1.013, 1.091]**	1.075 [1.019, 1.135]**
**Country of birth-air pollution interaction models**				
Born in UK (Reference)				
Not born in UK	1.023 [1.015, 1.032]**	1.241 [1.105, 1.393]**	1.050 [1.028, 1.073]**	1.077 [1.042, 1.112]**
No answer	1.00 [0.990, 1.010]	0.990 [0.907, 1.080]	0.988 [0.969, 1.008]	0.985 [0.957, 1.013]
	**Within pollutant effect**			
**Ethnicity-air pollution interaction models**	NO_2_ (μg/m^3^)	SO_2_ (μg/m^3^)	PM10 (μg/m^3^)	PM2.5 (μg/m^3^)
	OR [95%CI]	OR [95%CI]	OR [95%CI]	OR [95%CI]
British-white (Reference)				
Other-white	0.985 [0.912, 1.065]	1.536 [1.147, 2.055]**	1.00 [0.888, 1.126]	0.974 [0.833, 1.137]
Indian	1.004 [0.922, 1.094]	0.863 [0.58, 1.285]	0.946 [0.812, 1.103]	0.857 [0.69, 1.063]
Pakistani/Bangladeshi	1.044 [0.969, 1.124]	1.105 [0.799, 1.53]	1.079 [0.947, 1.229]	0.956 [0.789, 1.159]
Black/African/Caribbean	0.953 [0.877, 1.037]	1.220 [0.826, 1.802]	0.975 [0.83, 1.146]	0.971 [0.777, 1.213]
Mixed ethnicities	0.961 [0.852, 1.083]	0.477 [0.264, 0.86]*	0.699 [0.561, 0.871]**	0.640 [0.467, 0.877]**
Other ethnicities	0.923 [0.838, 1.017]	1.088 [0.718, 1.647]	0.913 [0.774, 1.076]	1.003 [0.803, 1.254]
**Country of birth-air pollution interaction models**				
Born in UK (Reference)				
Not born in UK	1.013 [0.965, 1.063]	1.230 [0.978, 1.548]	0.984 [0.906, 1.069]	0.912 [0.810, 1.026]
No answer	1.049 [1.003, 1.097]*	1.295 [1.069, 1.569]**	1.085 [1.019, 1.155]*	1.074 [0.983, 1.173]

**P-value<0.01

*P-value<0.05

ORs and 95%CIs are expressed in terms of 1 μg/m^3^ increase in the air pollutants; Air pollution-ethnicity interaction models are adjusted for country of birth, age, gender, marital status, education, perceived financial situation, socioeconomic classification, smoking status, year dummies (2015–2019), and rural-urban indicator; Air pollution-country of birth interaction models are adjusted for ethnicity, age, gender, marital status, education, perceived financial situation, socioeconomic classification, smoking status, year dummies (2015–2019), and rural-urban indicator.

**Table 6:** The overall and *Between-Within* effects of air pollution on all-cause outpatient hospital admissions by ethnicity and country of birth (*N* = 140,466 surveys from 46,442 individuals).

	**Overall pollutant effect**			
**Ethnicity-air pollution interaction models**	NO_2_ (μg/m^3^)	SO_2_ (μg/m^3^)	PM10 (μg/m^3^)	PM2.5 (μg/m^3^)
	OR [95%CI]	OR [95%CI]	OR [95%CI]	OR [95%CI]
British-white (Reference)				
Other-white	0.992 [0.981, 1.002]	1.037 [0.933, 1.152]	0.991 [0.968, 1.015]	0.98 [0.947, 1.014]
Indian	0.996 [0.978, 1.015]	1.021 [0.862, 1.209]	1.013 [0.972, 1.056]	1.014 [0.952, 1.081]
Pakistani/Bangladeshi	1.003 [0.99, 1.016]	1.123 [0.96, 1.313]	1.013 [0.984, 1.043]	1.02 [0.973, 1.07]
Black/African/Caribbean	1.001 [0.985, 1.017]	0.999 [0.84, 1.187]	1.046 [1.005, 1.088]*	1.065 [1.001, 1.133]*
Mixed ethnicities	0.998 [0.979, 1.018]	0.997 [0.787, 1.262]	1.00 [0.953, 1.05]	1.001 [0.93, 1.077]
Other ethnicities	0.995 [0.981, 1.009]	0.955 [0.808, 1.13]	1.013 [0.978, 1.048]	1.015 [0.966, 1.067]
**Country of birth-air pollution interaction models**				
Born in UK (Reference)				
Not born in UK	0.999 [0.991, 1.007]	0.982 [0.897, 1.076]	1.027 [1.008, 1.047]**	1.033 [1.004, 1.063]*
No answer	0.989 [0.98, 0.998]*	0.967 [0.902, 1.037]	0.986 [0.969, 1.003]	0.975 [0.951, 0.999]*
	**Between pollutant effect**			
**Ethnicity-air pollution interaction models**	NO_2_ (μg/m^3^)	SO_2_ (μg/m^3^)	PM10 (μg/m^3^)	PM2.5 (μg/m^3^)
	OR [95%CI]	OR [95%CI]	OR [95%CI]	OR [95%CI]
British-white (Reference)				
Other-white	0.993 [0.982, 1.004]	1.083 [0.955, 1.228]	0.993 [0.968, 1.019]	0.988 [0.952, 1.024]
Indian	1.001 [0.981, 1.021]	0.944 [0.765, 1.165]	1.036 [0.988, 1.086]	1.050 [0.976, 1.129]
Pakistani/Bangladeshi	1.005 [0.991, 1.019]	1.099 [0.886, 1.363]	1.022 [0.989, 1.055]	1.024 [0.972, 1.079]
Black/African/Caribbean	1.015 [0.998, 1.033]	0.977 [0.776, 1.23]	1.058 [1.013, 1.105]*	1.098 [1.024, 1.177]**
Mixed ethnicities	0.997 [0.976, 1.017]	0.945 [0.702, 1.273]	0.997 [0.946, 1.051]	0.994 [0.917, 1.078]
Other ethnicities	0.997 [0.983, 1.012]	0.950 [0.778, 1.161]	1.022 [0.986, 1.061]	1.026 [0.973, 1.083]
**Country of birth-air pollution interaction models**				
Born in UK (Reference)				
Not born in UK	1.003 [0.995, 1.011]	0.925 [0.826, 1.035]	1.033 [1.011, 1.055]**	1.044 [1.011, 1.077]**
No answer	0.988 [0.979, 0.997]*	0.934 [0.859, 1.016]	0.982 [0.963, 1.001]	0.973 [0.947, 1.00]
	**Within pollutant effect**			
**Ethnicity-air pollution interaction models**	NO_2_ (μg/m^3^)	SO_2_ (μg/m^3^)	PM10 (μg/m^3^)	PM2.5 (μg/m^3^)
	OR [95%CI]	OR [95%CI]	OR [95%CI]	OR [95%CI]
British-white (Reference)				
Other-white	1.010 [0.93, 1.098]	1.090 [0.798, 1.489]	1.036 [0.914, 1.176]	1.012 [0.859, 1.193]
Indian	0.996 [0.904, 1.096]	1.143 [0.735, 1.778]	0.955 [0.805, 1.133]	0.786 [0.617, 1.001]
Pakistani/Bangladeshi	1.056 [0.973, 1.146]	0.772 [0.538, 1.107]	0.864 [0.746, 1.001]	0.683 [0.549, 0.85]**
Black/African/Caribbean	0.96 [0.877, 1.051]	1.017 [0.668, 1.547]	0.927 [0.777, 1.106]	0.884 [0.693, 1.127]
Mixed ethnicities	1.069 [0.941, 1.215]	0.761 [0.407, 1.425]	0.847 [0.67, 1.07]	0.729 [0.522, 1.018]
Other ethnicities	0.931 [0.837, 1.036]	1.062 [0.674, 1.672]	0.922 [0.769, 1.106]	0.866 [0.678, 1.106]
**Country of birth-air pollution interaction models**				
Born in UK (Reference)				
Not born in UK	0.996 [0.945, 1.049]	0.915 [0.712, 1.174]	0.932 [0.851, 1.02]	0.838 [0.737, 0.953]**
No answer	1.02 [0.972, 1.07]	0.962 [0.786, 1.179]	1.004 [0.939, 1.074]	0.958 [0.871, 1.053]

**P-value<0.01

*P-value<0.05

ORs and 95%CIs are expressed in terms of 1 μg/m^3^ increase in the air pollutants; Air pollution-ethnicity interaction models are adjusted for country of birth, age, gender, marital status, education, perceived financial situation, socioeconomic classification, smoking status, year dummies (2015–2019), and rural-urban indicator; Air pollution-country of birth interaction models are adjusted for ethnicity, age, gender, marital status, education, perceived financial situation, socioeconomic classification, smoking status, year dummies (2015–2019), and rural-urban indicator.

Non-UK-born individuals were more likely to visit a GP than UK-born individuals with increasing concentrations of all the four pollutants (Table 5) and were more likely to go to a hospital with higher exposure to PM10 and PM2.5 (Table 6). It should be noted that in this study, ethnicity was examined separately from country of birth. Thus, we did not distinguish between non-UK-born versus UK-born within the same ethnicity, but rather we considered ethnicity and country of birth as two separate variables.

Analysing the *Between-Within* (spatial-temporal) effects of air pollution on all-cause GP visits and hospital admissions by ethnicity and country of birth showed some significant associations for the *Between* and *Within* effects. For example, a significant positive *Between* effect on GP visits was observed for SO_2_ pollutant among Pakistani/Bangladeshi individuals. A significant positive *Within* effect on GP visits was observed for SO_2_ pollutant among other-white individuals. Black/African/Caribbean ethnicities showed higher odds of outpatient hospital admissions with increasing concentrations of PM10 and PM2.5 for the *Between* effect compared to British-white (Tables 5 and 6).

Non-UK-born individuals also showed higher odds of GP visits with increasing concentrations of all the four pollutants for the *Between* (spatial) effects in comparison to UK-born individuals. Similarly, non-UK-born individuals showed higher odds of outpatient hospital admissions for the *Between* effects of PM10 and PM2.5 compared to UK-born individuals (Tables 5 and 6).

## Discussion

In this study, we showed that long-term exposure to four ambient air pollutants (NO_2_, SO_2_, PM10, and PM2.5) is associated with a higher frequency of all-cause GP visits and outpatient hospital admissions in the UK, which is supported by relevant literature [2, 8, 9, 69]. For instance, long-term exposure to NO_2_ and PM2.5 pollutants was associated with higher rates of hospital admissions for circulatory, cancer, and respiratory tract diseases in Italy [70]. In Australia, a 1% increase in PM10 was associated with a 0.6% increase in the number of hospital admissions and a 0.5% increase in the number of GP visits [71].

The application of a *Between-Within* analysis in this study revealed significant *Between* (spatial) effects for air pollution on all-cause GP visits and hospital admissions; yet, no significant *Within* (temporal) effects were noticed. Therefore, individuals residing in LSOAs with higher exposure to outdoor air pollution across the five years of follow-up exhibited higher frequency of GP visits and outpatient hospital admissions than individuals residing in LSOAs with lower pollution exposure. However, the yearly variation of air pollution across the five years of follow-up within each LSOA did not have an effect on all-cause GP visits and hospital admissions. Thus, our study shows strong evidence for the spatial rather than temporal effects of air pollution on GP visits and outpatient hospital admissions in the UK. Additionally, we observed a significant positive association between higher exposure to PM10 and PM2.5 pollutants and increased odds of GP visits when we accounted for Getis–Ord (Gi*) spatial autocorrelation measure, which was not the case when spatial autocorrelation was not accounted for. This provides further support for the spatial effects of air pollution on GP visits.

The fact of why we observed significant *Between* (spatial) but not *Within* (temporal) effects could be explained by the emission source of air pollutants and their chemical and physical properties. The major source of NO_2_ emissions is traffic exhaust [72] which varies across both LSOAs (*Between*: *spatial*) and time (*Within*: *temporal*) depending on the number of vehicles and the movement of people. Yet, nitrogen oxides are highly reactive and seasonal pollutants [73], which makes it difficult to capture their temporal variation through yearly measurements. For instance, more NO_2_ will be liberated into the atmosphere during warm seasons due to the chemical reactions between nitrogen oxides and ozone [73]. Additionally, NO_2_ is converted into nitric acid by several different reactions in the atmosphere [74]. That may be why only spatial (*Between*) but not temporal (*Within*) effects for NO_2_ pollutant were observed when taking the year as our time measuring unit. On the other hand, industrial processes and power plants are the major sources of SO_2_ pollution [75], which is dominated by spatial (*Between*) variation rather than temporal (*Within*) variation as building a new factory requires much longer time than purchasing a motor vehicle. Particulate matter results from both traffic exhaust and industrial processes [76], and is considered a more stable pollutant that may stay suspended in the air for long periods of time [74]. Given the localized industrial source of particulate matter pollution and its stability in the atmosphere which limits its variation across time, a spatial (*Between*) effect on health-related outcomes is expected rather than a temporal (*Within*) effect.

Furthermore, some of the observed *Between* effects may be related to contextual (e.g., area deprivation and the percentage of ethnic groups by area) rather than individual-level confounders. Our models accounted for various individual-level confounders and for urbanicity, but not for contextual LSOAs-level confounders, which may introduce ecological bias. Adjusting the models for contextual LSOAs-level confounders such as the multiple deprivation deciles would be a crude approach given that the UKHLS dataset covers the four nations of the UK (i.e., England, Wales, Scotland, and Northern Ireland), and each of the four nations have their own criteria and indicators for calculating the multiple deprivation indexes, which reduces comparability [77–79]. In addition, these deprivation indexes are published on different timescales and cover different time periods, which makes it difficult to link them to longitudinal datasets. Adjusting the models for the percentages of ethnic groups by LSOAs would also lead to biased results because these data are only collected once every ten years during the census [80].

As for the insignificant *Within* effects for all the pollutants, these could be attributed to the relatively short period of follow-up (2015–2019) and the fact that yearly air pollution data were used rather than monthly or daily data. This limits the temporal variation in the air pollutant concentrations and reduces the statistical power needed to observe significant *Within* effects. For example, the assessment of temporal effects in a study conducted in South London by calculating the difference between daily rather than yearly air pollutant concentrations and the five years mean for each pollutant per LSOA revealed a higher temporal than spatial variability for PM10, PM2.5 and ozone pollutants [81].

To sum up, several factors could have played a role in the current study to explain why a spatial effect was more apparent than a temporal one. Those include (1) the emission source of air pollutants and their chemical and physical properties, (2) the short period of time studied in comparing the different years, (3) the modelling approach in which more (otherwise unmeasured, non-time-varying) confounders are held constant in the within approach, and (4) the limited variation in the air pollutants concentrations between years in most locations, thus giving inadequate power to detect a *Within* effect.

This study also contributed to the topic of ethnic inequalities in health by investigating the differences in the effect of air pollution on all-cause GP visits and outpatient hospital admissions by ethnicity and country of birth in the UK. Our results showed no support for ethnic differences. However, non-UK-born individuals were more likely to visit a GP and go to a hospital than UK-born individuals with increasing concentrations of air pollutants. This was attributed to spatial area-specific rather than time-specific factors as shown in the *Between-Within* analysis.

Research has shown that immigrants (i.e. non-UK-born) often live in large cities, near major roads and key transportation networks. This facilitates their movement and increases their chances of personal development, employment, and business start-ups [82]. Furthermore, immigrants often live in low-priced housing, which is often situated in more deprived immigrants’ concentrated neighbourhoods or close to major roads [38]. These area-specific factors can result in more pronounced mild health consequences and higher frequency of GP visits and outpatient hospital admissions among immigrants due to the greater exposure to traffic exhaustion and industrial pollution in comparison to UK-born individuals who have additional financial resources to move away from metropolitan areas and highly polluted industrial regions. Additionally, immigrants (especially those coming from outside Europe) might have been exposed to higher air pollution concentrations in their countries of origin prior to arriving in the UK, which might result in higher frequency of GP visits and outpatient hospital admissions due to the cumulative effect of air pollution on health among non-UK-born individuals compared to UK-born.

In addition to highlighting the contributions of the present study, it is equally important to discuss its limitations. First, this study might been subject to exposure bias by considering the individual’s exposure to outdoor air pollution only at the place of residence. Therefore, future studies are encouraged to integrate air pollution exposures at the places of residence and work, and across daily commuting patterns by using for example real-time GPS data of the exact location of individuals. Second, this study assessed the association between long-term exposure to outdoor air pollution and all-cause self-reported GP visits and outpatient hospital admissions. Self-reported survey data are prone to recall and information bias [83]. Nevertheless, there is literature which validate the accuracy of the UKHLS GP visits and outpatient hospital admissions by comparing those two variables to the administrative data for GP and outpatient consultations in England and Scotland, whereby the mean counts between the two datasets were reasonably close with some evidence of moderate under-reporting in the UKHLS data [84]. Thus, future research would further contribute to this topic by investigating all-cause and cause-specific GP-visits and hospital admissions using data from administrative-based studies such as the ONS Longitudinal Study for England and Wales and the Scottish Longitudinal Study for Scotland. Third, the association between air pollution and GP visits and hospital admissions by country of birth was assessed using a binary variable that combines non-UK-born individuals in one group and compares them to UK-born individuals. Whilst this approach can reveal the differences between the two sub-population groups, it cannot provide detailed insights into the differences between the non-UK-born group itself based on the country of origin. Thus, disaggregation by immigrant sub-group is recommended for future studies for an indepth understanding of ethnic inequalities related to deprivation, health disadvantage, health needs, and access to healthcare [85]. For example, literature from the UK has shown that immigrants from certain countries (e.g., South Asia and Sub-Saharan Africa) have a significant burden of infectious diseases [86, 87], which could lead to higher rates of GP visits and hospital admissions. On the other hand, immigrants from certain countries, especially undocumented immigrants and asylum seekers might face challenges to access primary healthcare [88, 89], which reduces their frequency of hospital admissions and GP visits. However, it should be noted that in the present study, we adjusted the models which assess the association of air pollution with GP visits and hospital admissions by country of birth for ethnicity. This absorbs some of the uncertainty related to the binary immigration variable. Finally, our study included individuals followed over five years (2015–2019) and we assessed the association between air pollution and GP visits and hospital admissions at a yearly rather than monthly or daily basis, which did not allow for environmental seasonal factors (e.g., temperature and relative humidity) adjustment. This could also explain the inability of observing significant temporal (*Within*) effects of air pollution on GP visits and outpatient hospital admissions due to the low variation in air pollution across the five years of follow-up and the limited statistical power of yearly observations.

## Conclusion

This study supports an association between increased exposure to long-term outdoor air pollution and higher frequency of all-cause GP visits and outpatient hospital admissions in the UK. Residing in more polluted areas was the driving cause for more GP visits and hospital admissions rather than the fluctuation between low and high exposures to air pollution across time. The observed associations did not differ across ethnic groups, but by country of birth, they were more pronounced in individuals born outside UK than those born in UK. These results are of importance for policymakers to reduce air pollution emissions, which would lower the burden on GPs and hospital care in the UK. Further longitudinal studies with longer follow-up time periods may be able to reveal more definite conclusions on the influence of ethnicity on the association between long-term outdoor air pollution and both hospital admissions and GP visits.

## Supporting information

S1 File (PDF)

S2 File (PDF)
