# Supplementary material for: The association of long-term exposure to outdoor air pollution with all-cause GP visits and hospital admissions by ethnicity and country of birth in the United Kingdom
Source: PLoS One. 2023 Oct 11;18(10):e0275414. doi: 10.1371/journal.pone.0275414 (PMC10566689; doi:10.1371/journal.pone.0275414)
Supplement: S1 File — (PDF) [file pone.0275414.s001.pdf]

Supplementary File 1. Stepwise modelling and model validation estimates

Table 1: Description of stepwise models 1 to 5

	Outcome	Independent variable	Covariates
Model 1	GP visits or Outpatient hospital admissions	NO_2 or SO_2 or PM10 or PM2.5	Age, gender, and year dummies
Model 2	GP visits or Outpatient hospital admissions	NO_2 or SO_2 or PM10 or PM2.5	Age, gender, year dummies, ethnicity, and country of birth
Model 3	GP visits or Outpatient hospital admissions	NO_2 or SO_2 or PM10 or PM2.5	Age, gender, year dummies, ethnicity, country of birth, marital status, education, perceived financial situation, and socioeconomic classification
Model 4	GP visits or Outpatient hospital admissions	NO_2 or SO_2 or PM10 or PM2.5	Age, gender, year dummies, ethnicity, country of birth, marital status, education, perceived financial situation, socioeconomic classification, and smoking status
Model 5	GP visits or Outpatient hospital admissions	NO_2 or SO_2 or PM10 or PM2.5	Age, gender, year dummies, ethnicity, country of birth, marital status, education, perceived financial situation, socioeconomic classification, smoking status, and rural-urban indicator

Table 2: Stepwise models for the association of air pollution with all-cause GP visits and outpatient hospital admissions with AIC and BIC estimates (N=140,466 surveys from 46,442 individuals)

	GP visits<br>OR [95%CI]	Outpatient hospital admissions<br>OR [95%CI]
**Model 1 is adjusted for age, gender, and time dummies**		
NO_2 (µg/m^3)	1.023 [1.019, 1.026]**	1.008 [1.005, 1.011]**
SO_2 (µg/m^3)	1.220 [1,181, 1.260]**	1.086 [1.053, 1.119]**
PM10 (µg/m^3)	1.020 [1.013, 1.028]**	1.013 [1.006, 1.020]**
PM2.5 (µg/m^3)	1.031 [1.020, 1.041]**	1.021 [1.011, 1.031]**
AIC - NO_2	361446.5	302152.5
BIC - NO_2	361692.8	302398.8
AIC - SO_2	361470.6	302153.3
BIC - SO_2	361716.9	302399.6
AIC – PM10	361586.5	302167.6
BIC – PM10	361832.8	302413.9
AIC – PM2.5	361581.9	302163.0
BIC – PM2.5	361828.2	302409.3
**Model 2 = Model 1 + Ethnicity and country of birth**		
NO_2 (µg/m^3)	1.013 [1.009, 1.016]**	1.010 [1.007, 1.014]**
SO_2 (µg/m^3)	1.171 [1.133, 1.210]**	1.089 [1.056, 1.124]**
PM10 (µg/m^3)	1.000 [0.993, 1.008]	1.013 [1.006, 1.020]**
PM2.5 (µg/m^3)	1.002 [0.991, 1.013]	1.022 [1.011, 1.032]**
AIC - NO_2	361243.5	302118.1
BIC - NO_2	361568.7	302443.2
AIC - SO_2	361196.6	302121.3
BIC - SO_2	361521.8	302446.5
AIC – PM10	361286.5	302138.4
BIC – PM10	361611.6	302463.5
AIC – PM2.5	361286.4	302133.3
BIC – PM2.5	361611.5	302458.5
**Model 3 = Model 2 + marital status, education, perceived financial situation, and socioeconomic classification**		
NO_2 (µg/m^3)	1.012 [1.008, 1.015]**	1.010 [1.006, 1.013]**
SO_2 (µg/m^3)	1.128 [1.093, 1.165]**	1.067 [1.035, 1.101]**
PM10 (µg/m^3)	1.003 [0.996, 1.011]	1.014 [1.007, 1.022]**
PM2.5 (µg/m^3)	1.006 [0.995, 1.017]	1.023 [1.013, 1.034]**
AIC - NO_2	359386.9	301162.6
BIC - NO_2	359859.8	301635.6
AIC - SO_2	359369.6	301175.2
BIC - SO_2	359842.6	301648.1
AIC – PM10	359424.0	301178.0
BIC – PM10	359896.9	301650.9
AIC – PM2.5	359423.6	301172.9
BIC – PM2.5	359896.6	301645.8
**Model 4 = Model 3 + smoking status**		

NO_2 (μg/m^3)	1.011 [1.008, 1.015]**	1.010 [1.006, 1.013]**
SO_2 (μg/m^3)	1.127 [1.091, 1.163]**	1.067 [1.035, 1.101]**
PM10 (μg/m^3)	1.003 [0.996, 1.011]	1.014 [1.007, 1.022]**
PM2.5 (μg/m^3)	1.006 [0.995, 1.017]	1.023 [1.013, 1.034]**
AIC - NO_2	359369.4	301164.2
BIC - NO_2	359862.0	301656.8
AIC - SO_2	359353.0	301176.7
BIC - SO_2	359845.6	301669.4
AIC – PM10	359406.0	301179.5
BIC – PM10	359898.6	301672.2
AIC – PM2.5	359405.6	301174.4
BIC – PM2.5	359898.3	301667.0
**Model 5 = Model 4 + rural-urban indicator**		
NO_2 (μg/m^3)	1.010 [1.006, 1.014]**	1.008 [1.004, 1.012]**
SO_2 (μg/m^3)	1.114 [1.077, 1.152]**	1.048 [1.014, 1.083]**
PM10 (μg/m^3)	0.999 [0.992, 1.007]	1.011 [1.003, 1.018]**
PM2.5 (μg/m^3)	0.999 [0.987, 1.010]	1.018 [1.007, 1.029]**
AIC - NO_2	359369.0	301162.1
BIC - NO_2	359871.5	301664.6
AIC - SO_2	359351.0	301168.6
BIC - SO_2	359853.5	301671.1
AIC – PM10	359390.4	301168.6
BIC – PM10	359892.8	301671.1
AIC – PM2.5	359390.3	301166.3
BIC – PM2.5	359892.8	301668.8

**P-value <0.01; *P-value<0.05; ORs and 95%CIs are expressed in terms of 1 μg/m^3 increase in the air pollutants.

Table 3: The association of GP visits with the socioeconomic and lifestyle covariates (N=140,466 surveys from 46,442 individuals)

		**OR**	**lower 95%CI**	**Upper 95% CI**	**P-value**
**Ethnicity**	British-white (Reference)				
	Other-white	0.84	0.76	0.93	0.001
	Indian	1.27	1.13	1.43	0.000
	Pakistani/Bangladeshi	1.49	1.34	1.66	0.000
	Black/African/Caribbean	1.08	0.97	1.21	0.167
	Mixed ethnicities	1.23	1.06	1.42	0.006
	Other ethnicities	0.87	0.77	0.98	0.027
**Country of birth**	Born in the UK (Reference)				
	Not born in the UK	1.23	1.14	1.33	0.000
	No answer	0.78	0.73	0.84	0.000
**Age**	16-18 (Reference)				
	19-23	1.11	1.01	1.22	0.031
	24-28	1.27	1.13	1.43	0.000
	29-33	1.31	1.16	1.48	0.000
	34-38	1.20	1.06	1.35	0.004
	39-43	1.26	1.11	1.42	0.000
	44-48	1.44	1.28	1.63	0.000
	49-53	1.61	1.43	1.82	0.000
	54-58	1.91	1.69	2.16	0.000
	59-63	1.97	1.74	2.23	0.000
	64-68	2.09	1.84	2.38	0.000
	69-73	2.32	2.03	2.64	0.000
	74-78	2.84	2.47	3.26	0.000
	>78	3.57	3.09	4.12	0.000
**Gender**	Male (Reference)				
	Female	2.24	2.15	2.33	0.000
**Education**	University degree (Reference)				
	High school degree	1.05	1.00	1.11	0.058
	Lower educational levels	1.51	1.24	1.83	0.000
	Other qualifications	1.45	1.36	1.54	0.000
	Still a student	0.80	0.73	0.88	0.000
**Marital status**	Married (Reference)				
	Living as a couple	1.14	1.07	1.21	0.000
	Widowed	0.91	0.84	1.00	0.040
	Divorced/separated	1.21	1.13	1.30	0.000
	Single never married	0.99	0.93	1.05	0.650
	No answer	0.91	0.71	1.16	0.444
**Subjective financial situation**	Living comfortably/doing alright (Reference)				
	Living difficultly	1.13	1.07	1.18	0.000
	No answer	0.96	0.69	1.34	0.814

**Socio-economic classification**	Management and professional occupations (Reference)				
	Intermediate occupations	1.50	1.45	1.55	0.000
	Routine occupations	0.82	0.65	1.03	0.091
	NA: student/retired/not working				
	No answer	0.99	0.94	1.05	0.850
**Smoking status**	Non-smoker (Reference)	1.12	1.06	1.18	0.000
	Smoker	1.84	1.75	1.95	0.000
	No answer	1.01	0.90	1.14	0.826
**Time dummies**	2015 (Reference)				
	2016	1.15	1.11	1.19	0.000
	2017	1.20	1.15	1.24	0.000
	2018	0.89	0.86	0.92	0.000
	2019	0.71	0.67	0.74	0.000
**Rural-Urban indicator**	Urban (Reference)				
	Rural	0.89	0.84	0.94	0.000

Table 4: The association of outpatient hospital admissions with the socioeconomic and lifestyle covariates (*N*=140,466 surveys from 46,442 individuals)

		OR	lower 95%CI	Upper 95% CI	P-value
**Ethnicity**	British-white (Reference)				
	Other-white	0.99	0.90	1.09	0.786
	Indian	0.89	0.79	0.99	0.038
	Pakistani/Bangladeshi	0.87	0.78	0.96	0.006
	Black/African/Caribbean	1.08	0.97	1.20	0.172
	Mixed ethnicities	1.36	1.18	1.57	0.000
	Other ethnicities	0.92	0.81	1.03	0.154
**Country of birth**	Born in the UK (Reference)				
	Not born in the UK	0.89	0.82	0.96	0.002
	No answer	0.95	0.89	1.02	0.159
**Age**	16-18 (Reference)				
	19-23	0.95	0.85	1.05	0.301
	24-28	1.10	0.97	1.25	0.123
	29-33	1.22	1.07	1.39	0.003
	34-38	1.13	1.00	1.29	0.059
	39-43	1.24	1.09	1.41	0.001
	44-48	1.36	1.20	1.55	0.000
	49-53	1.59	1.40	1.81	0.000
	54-58	1.94	1.71	2.21	0.000
	59-63	2.13	1.86	2.43	0.000
	64-68	2.30	2.01	2.63	0.000
	69-73	2.60	2.27	2.98	0.000
	74-78	3.31	2.87	3.82	0.000
	>78	3.83	3.30	4.44	0.000
**Gender**	Male (Reference)				
	Female	1.46	1.40	1.52	0.000
**Education**	University degree (Reference)				
	High school degree	1.00	0.95	1.06	0.964
	Lower educational levels	1.07	0.88	1.30	0.498
	Other qualifications	0.99	0.93	1.05	0.622
	Still a student	0.66	0.60	0.73	0.000
**Marital status**	Married (Reference)				
	Living as a couple	1.00	0.93	1.06	0.933
	Widowed	0.95	0.87	1.04	0.254
	Divorced/separated	1.23	1.14	1.31	0.000
	Single never married	0.90	0.85	0.96	0.001
	No answer	0.83	0.64	1.08	0.172
**Subjective financial situation**	Living comfortably/doing alright (Reference)				
	Living difficultly	1.00	0.95	1.05	0.934
	No answer	0.74	0.51	1.08	0.119

**Socio-economic classification**	Management and professional occupations (Reference)				
	Intermediate occupations	1.30	1.26	1.35	0.000
	Routine occupations	1.22	0.96	1.57	0.107
	NA: student/retired/not working				
	No answer	0.94	0.88	1.00	0.041
**Smoking status**	Non-smoker (Reference)	0.99	0.94	1.05	0.823
	Smoker	1.67	1.58	1.76	0.000
	No answer	1.05	0.93	1.19	0.419
**Time dummies**	2015 (Reference)				
	2016	1.09	1.05	1.14	0.000
	2017	1.15	1.11	1.20	0.000
	2018	1.05	1.01	1.10	0.017
	2019	1.00	0.95	1.05	0.958
**Rural-urban indicator**	Urban (Reference)				
	Rural	0.89	0.85	0.94	0.000

Table 5: The average yearly variations in air pollutants from the five years mean for each year between 2015 and 2019

	**Year**				
	**2015**	**2016**	**2017**	**2018**	**2019**
**NO_2 (μg/m^3)**	0.65	1.58	0.51	0.88	0.74
**SO_2 (μg/m^3)**	0.22	0.19	0.10	0.09	0.15
**PM10 (μg/m^3)**	0.68	0.75	0.56	0.40	0.58
**PM2.5 (μg/m^3)**	0.55	0.43	0.35	0.30	0.39

Table 6: The association of air pollution with all-cause GP visits and outpatient hospital admissions adjusting for spatial autocorrelation in air pollution using Getis-Ord Gi* measurement (*N*=140,466 surveys from 46,442 individuals)

	GP visits OR [95%CI]	Outpatient hospital admissions OR [95%CI]
NO_2 (μg/m^3)	1.011 [1.006, 1.017]**	1.011 [1.006, 1.017]**
SO_2 (μg/m^3)	1.093 [1.047, 1.142]**	1.038 [0.994, 1.084]
PM10 (μg/m^3)	1.019 [1.006, 1.032]**	1.028 [1.015, 1.041]**
PM2.5 (μg/m^3)	1.021 [1.002, 1.040]*	1.041 [1.022, 1.060]**

**P-value <0.01; *P-value<0.05;

ORs and 95%CIs are expressed in terms of 1 μg/m^3 increase in the air pollutants; Models are adjusted for age, gender, ethnicity, country of birth, marital status, education, perceived financial situation, socioeconomic classification, smoking status, year dummies (2015-2019), rural-urban indicator, and spatial autocorrelation in air pollution using Getis-Ord Gi* measurement.
