# Supplementary material for: The association of long-term exposure to outdoor air pollution with all-cause GP visits and hospital admissions by ethnicity and country of birth in the United Kingdom
Source: PLoS One. 2023 Oct 11;18(10):e0275414. doi: 10.1371/journal.pone.0275414 (PMC10566689; doi:10.1371/journal.pone.0275414)

Supplementary File 2. Maps showing the spatial autocorrelation in air pollution for each pollutant in each year between 2015 and 2019 across the LSOAs in the UK using Getis–Ord (Gi*) Cold-Hot spot analysis that employs a distance-based method of 5 Km ensuring that each LSOA has at least one neighbour.

Map 1. NO_2 pollutant in 2015

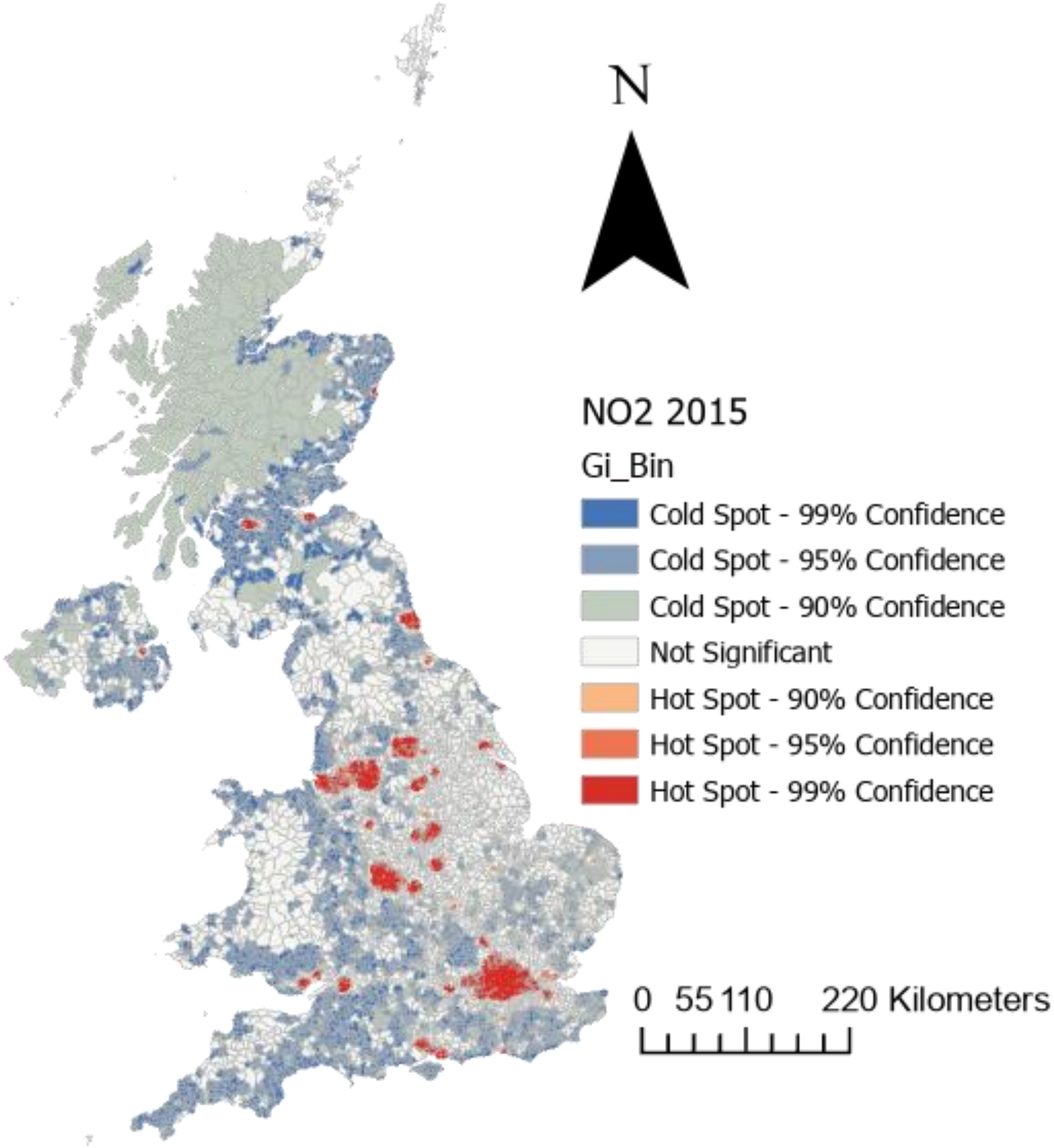

Map 2. NO_2 pollutant in 2016

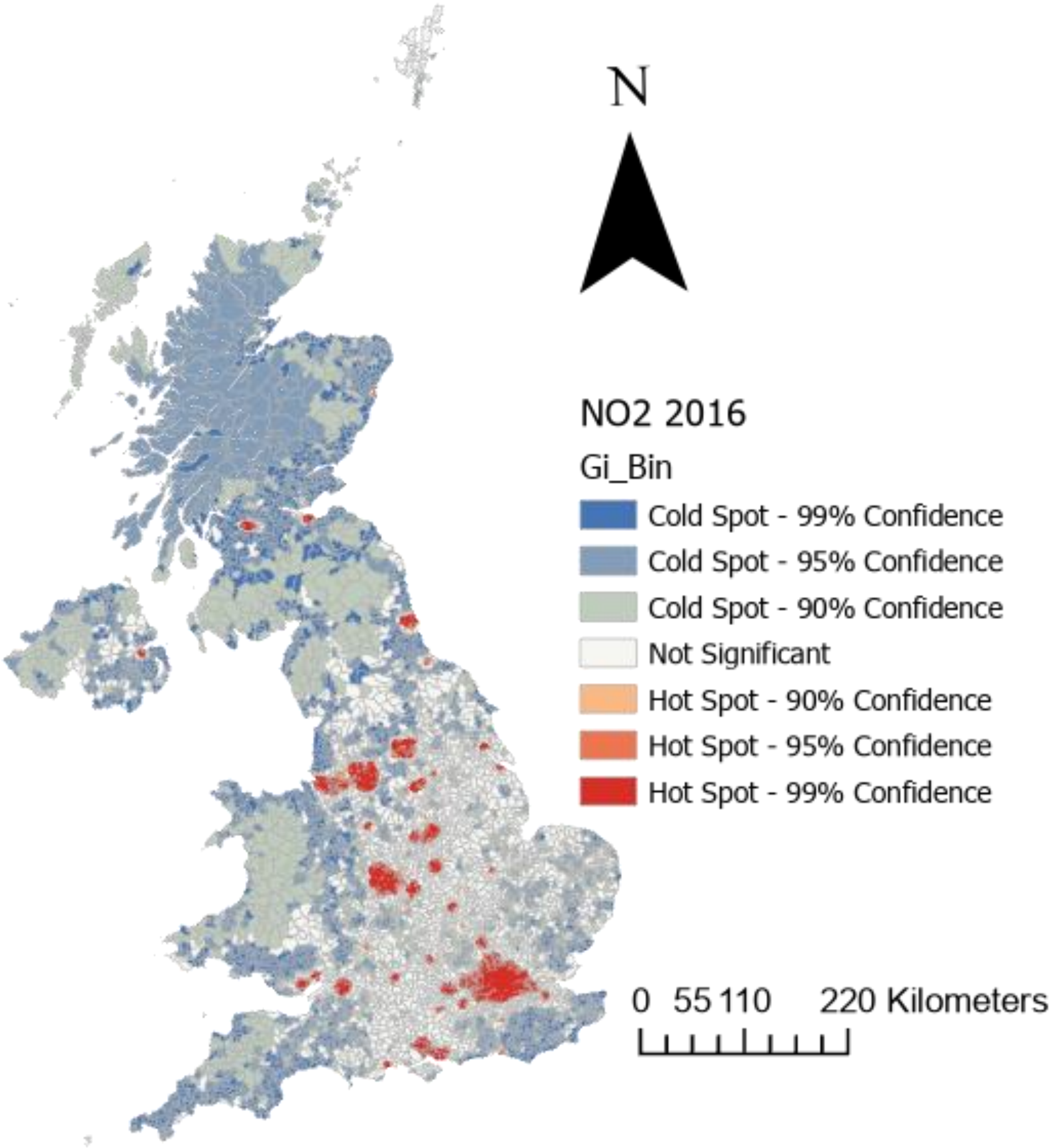

Map 3. NO_2 pollutant in 2017

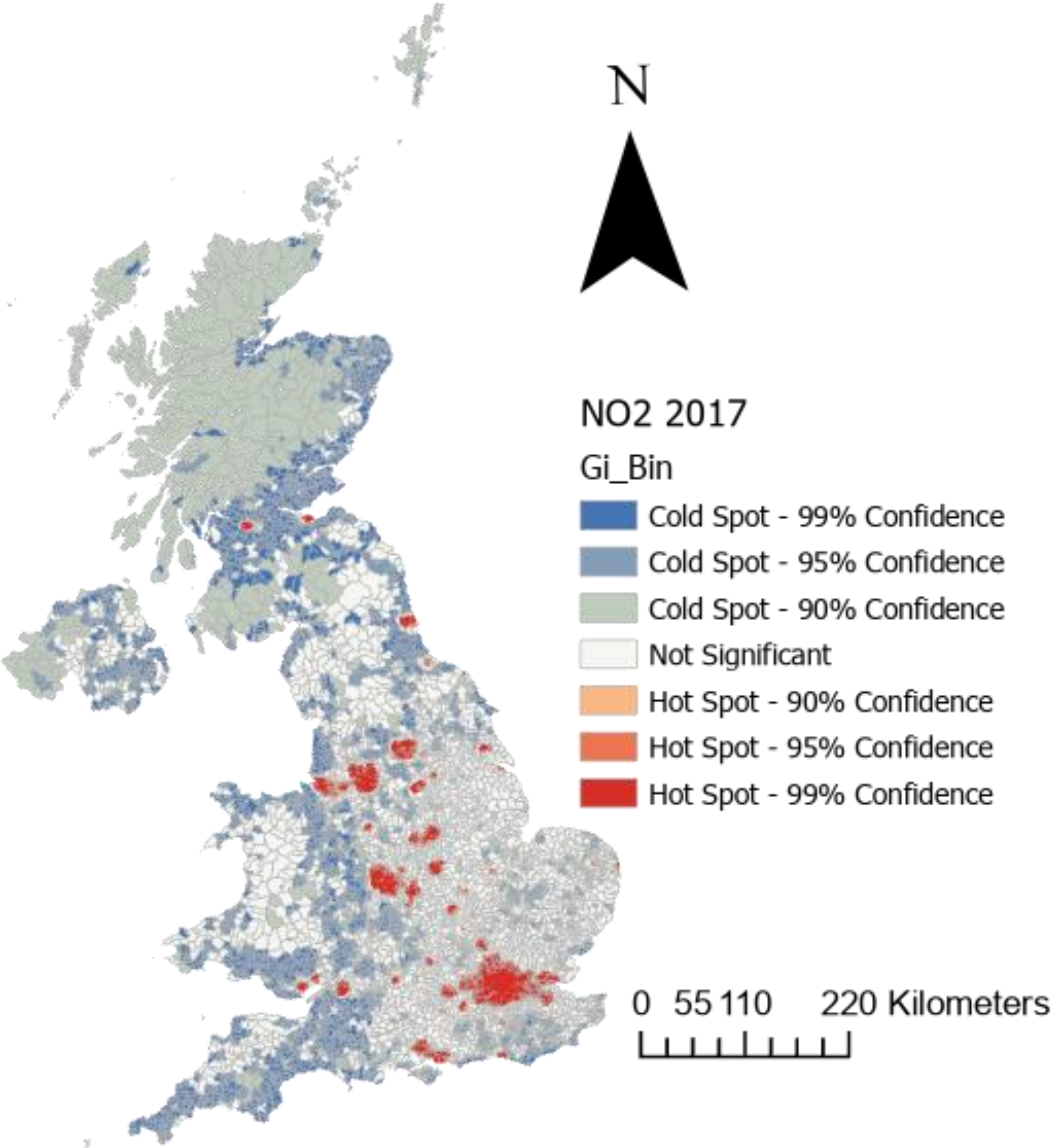

Map 4. NO_2 pollutant in 2018

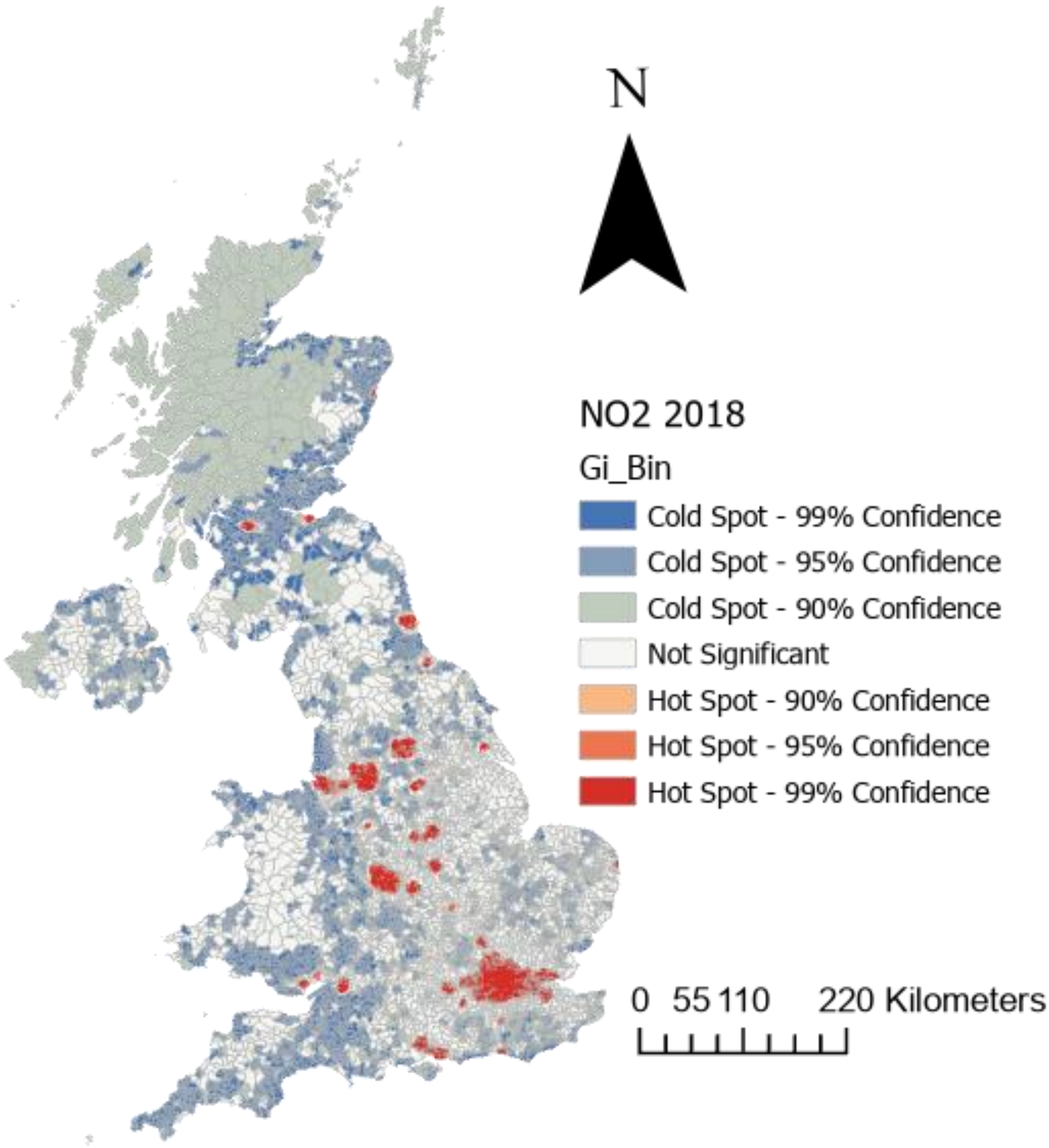

Map 5. NO_2 pollutant in 2019

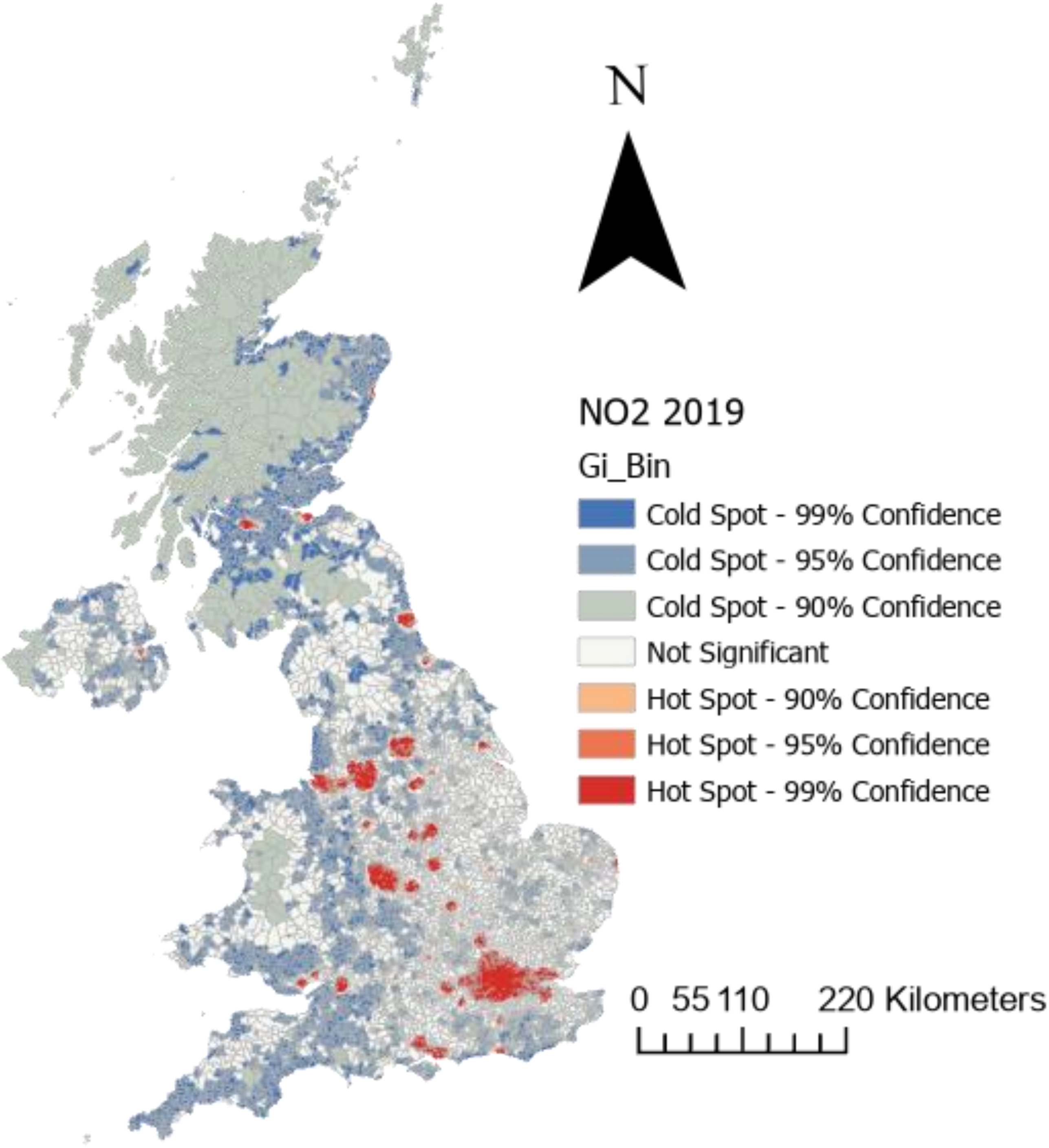

Map 6. SO_2 pollutant in 2015

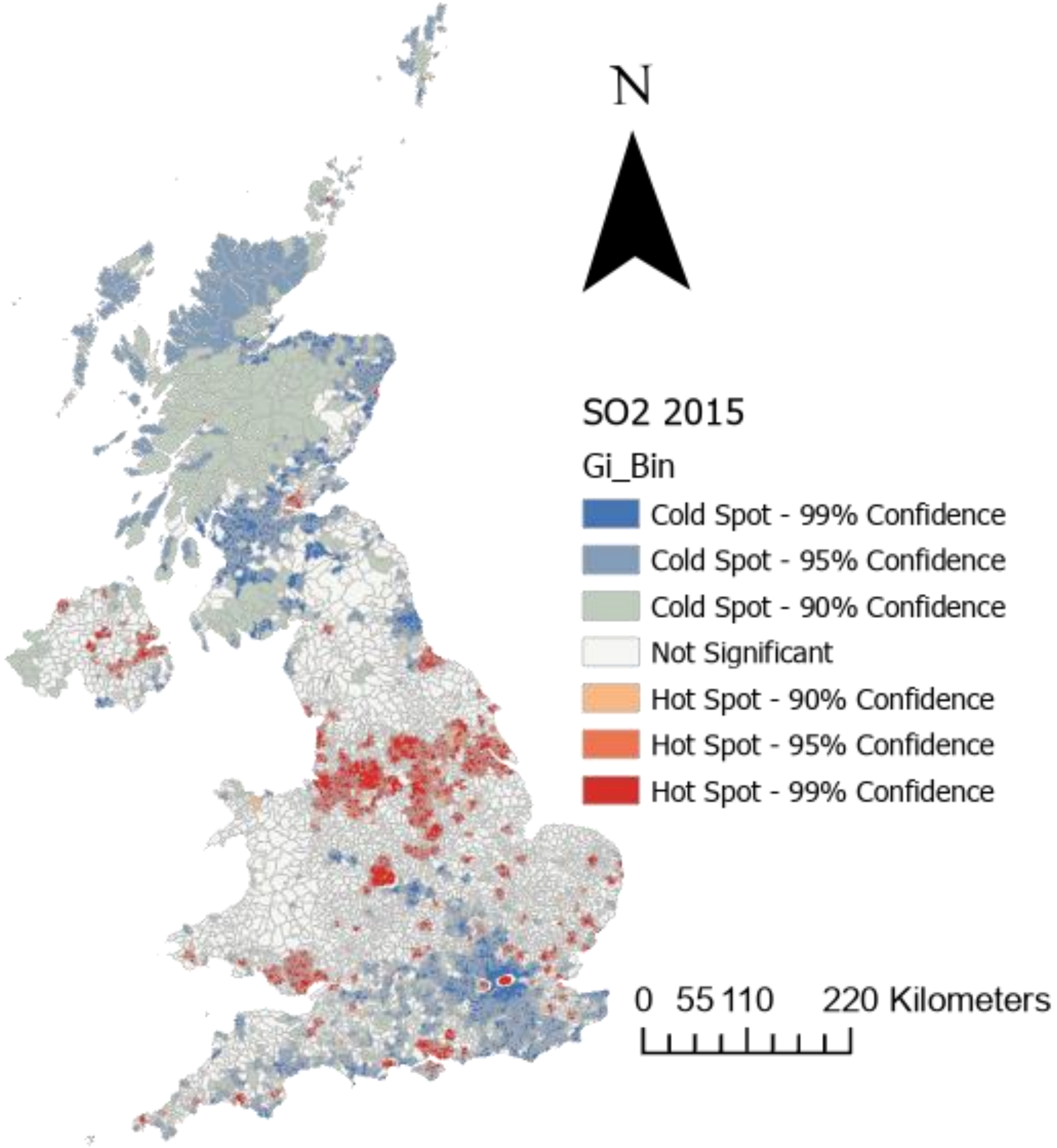

Map 7. SO_2 pollutant in 2016

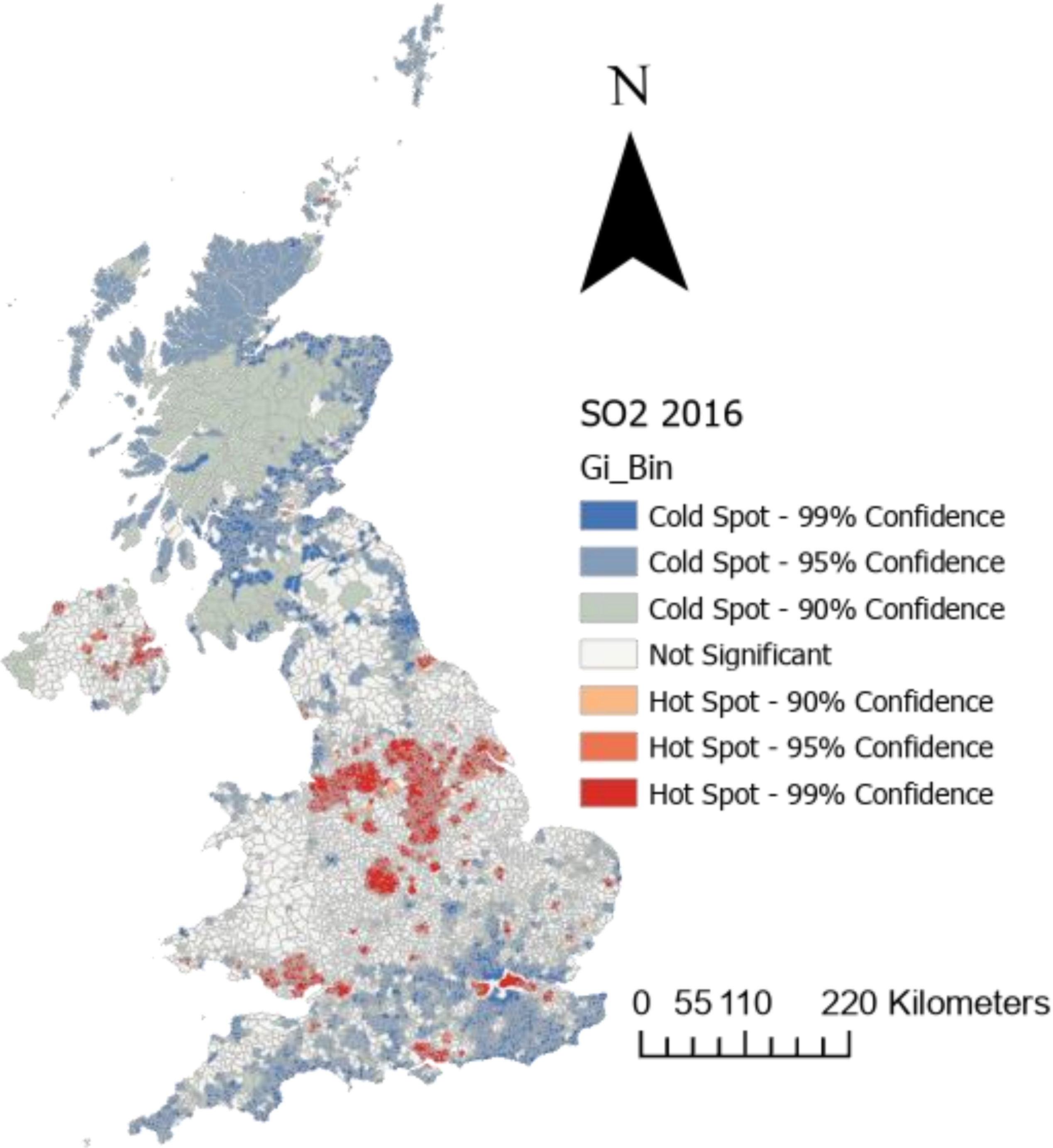

Map 8. SO_2 pollutant in 2017

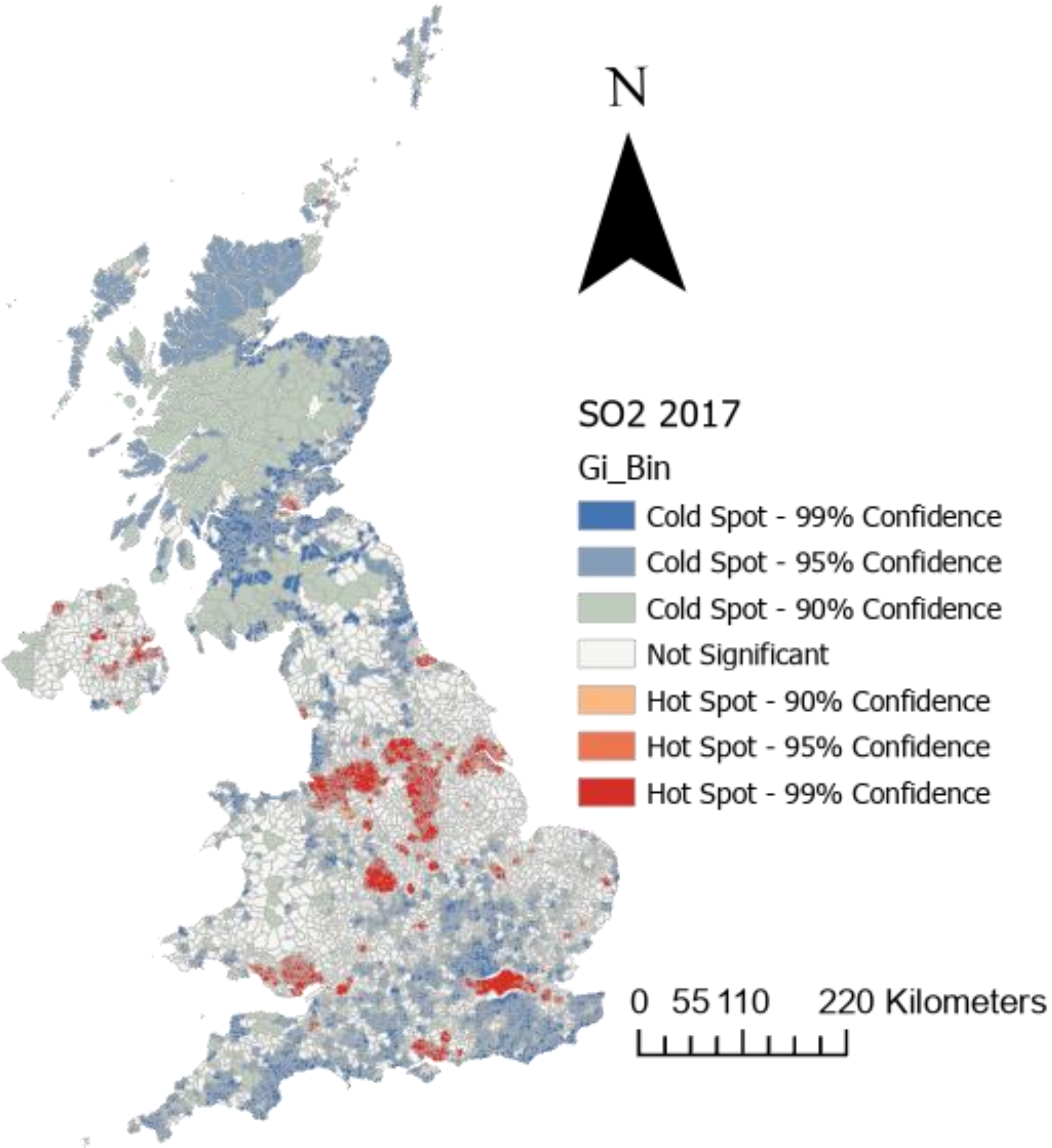

Map 9. SO_2 pollutant in 2018

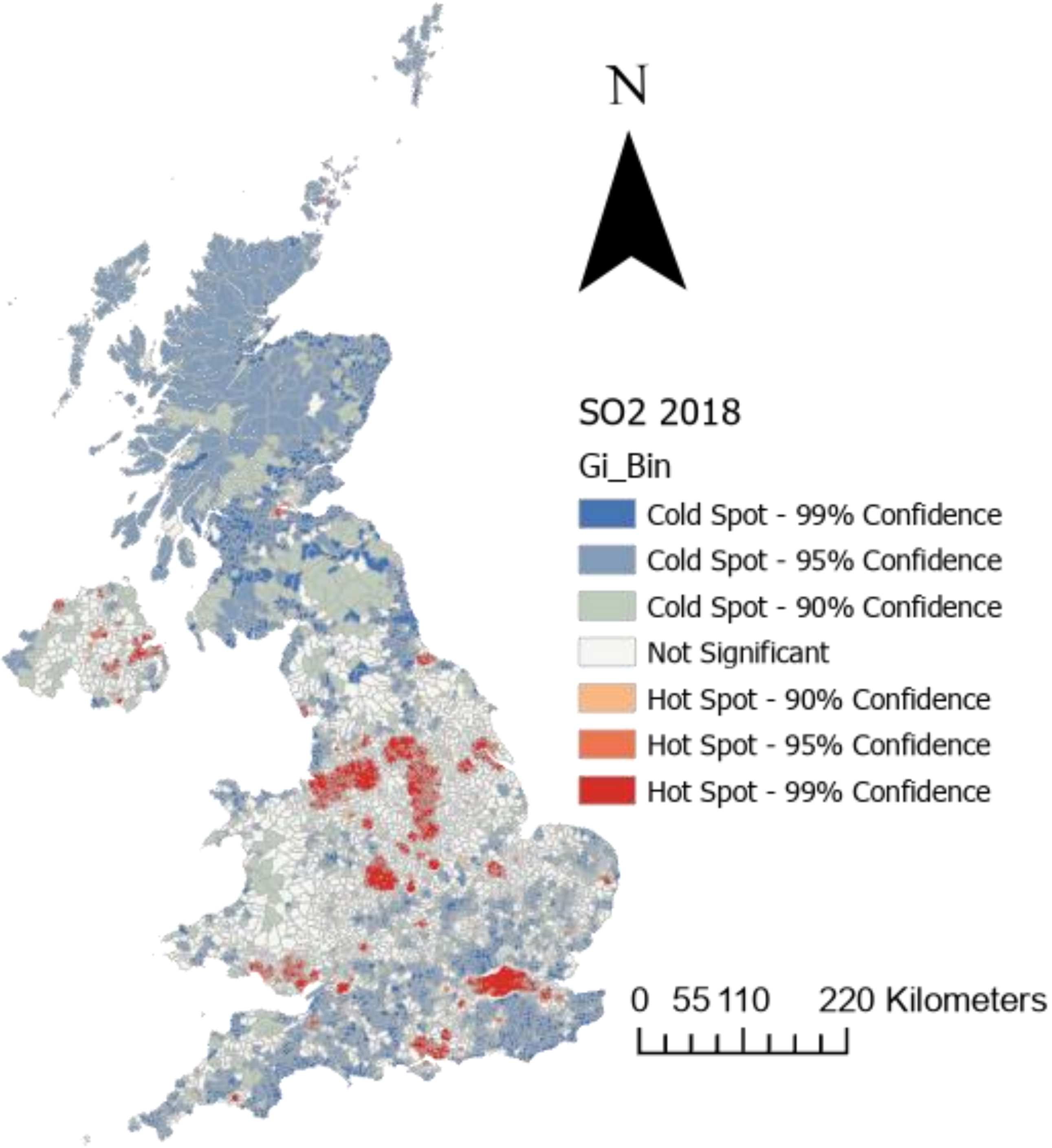

Map 10. **SO_2 pollutant in 2019**

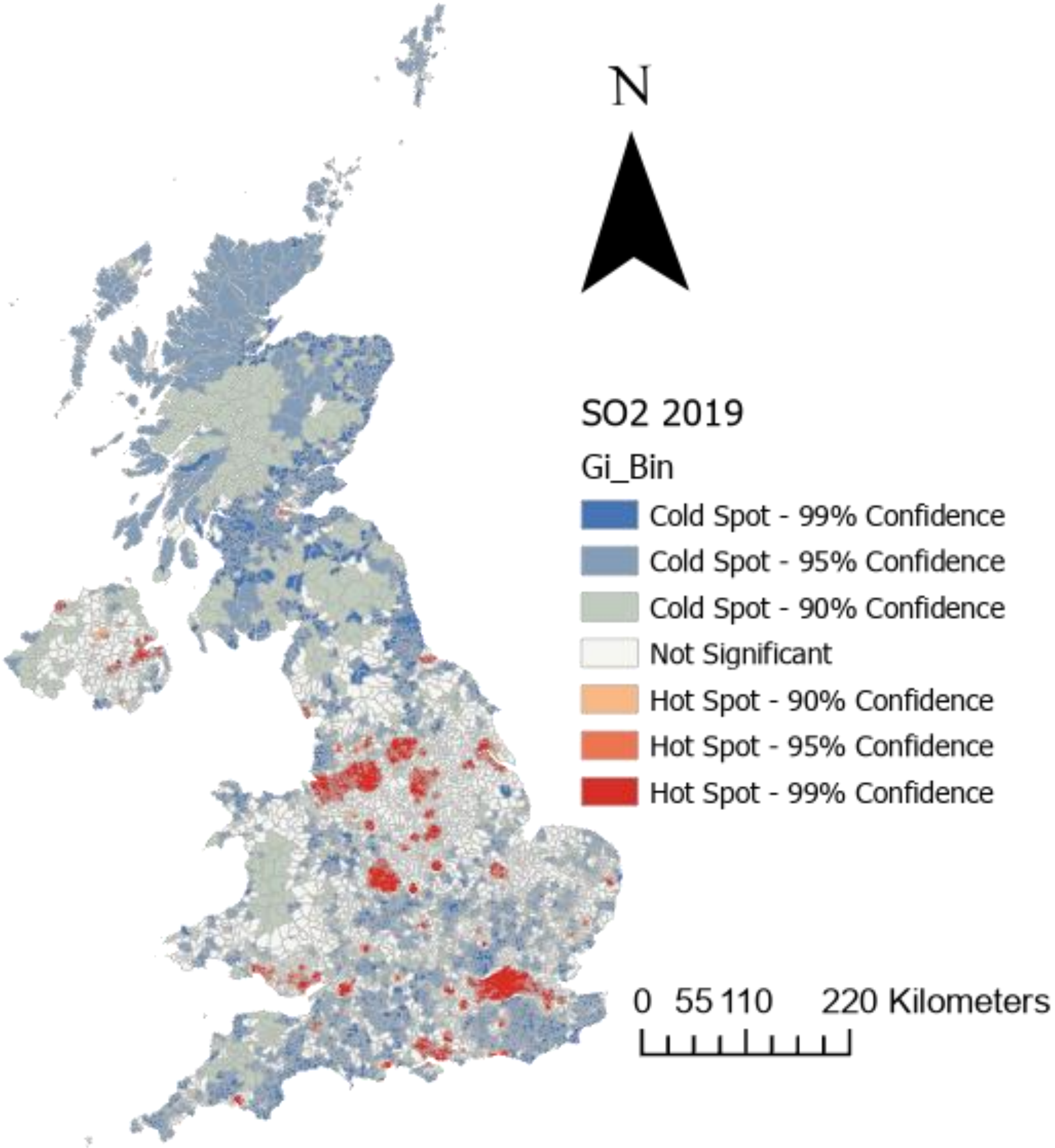

Map 11. PM10 pollutant in 2015

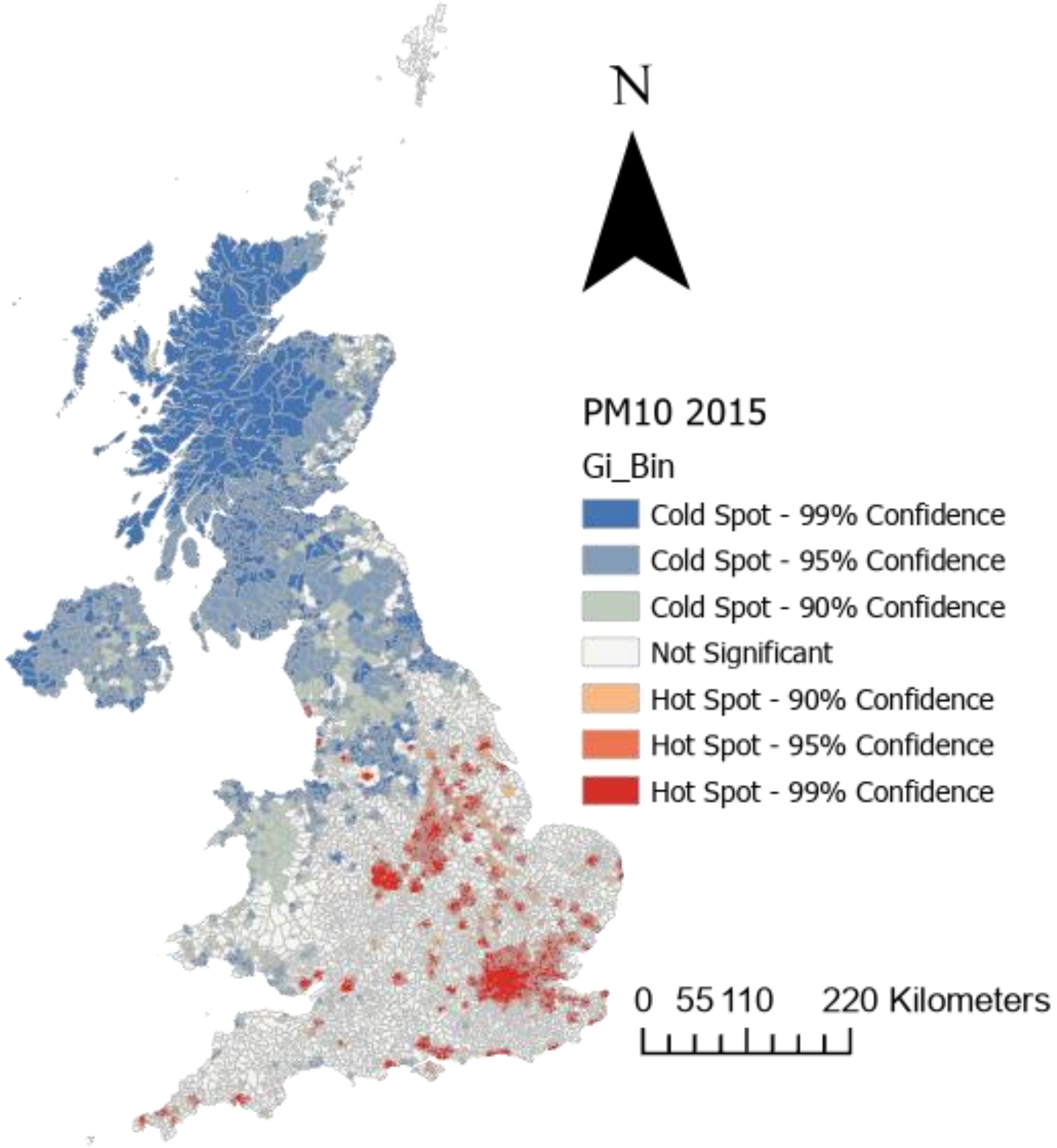

Map 12. PM10 pollutant in 2016

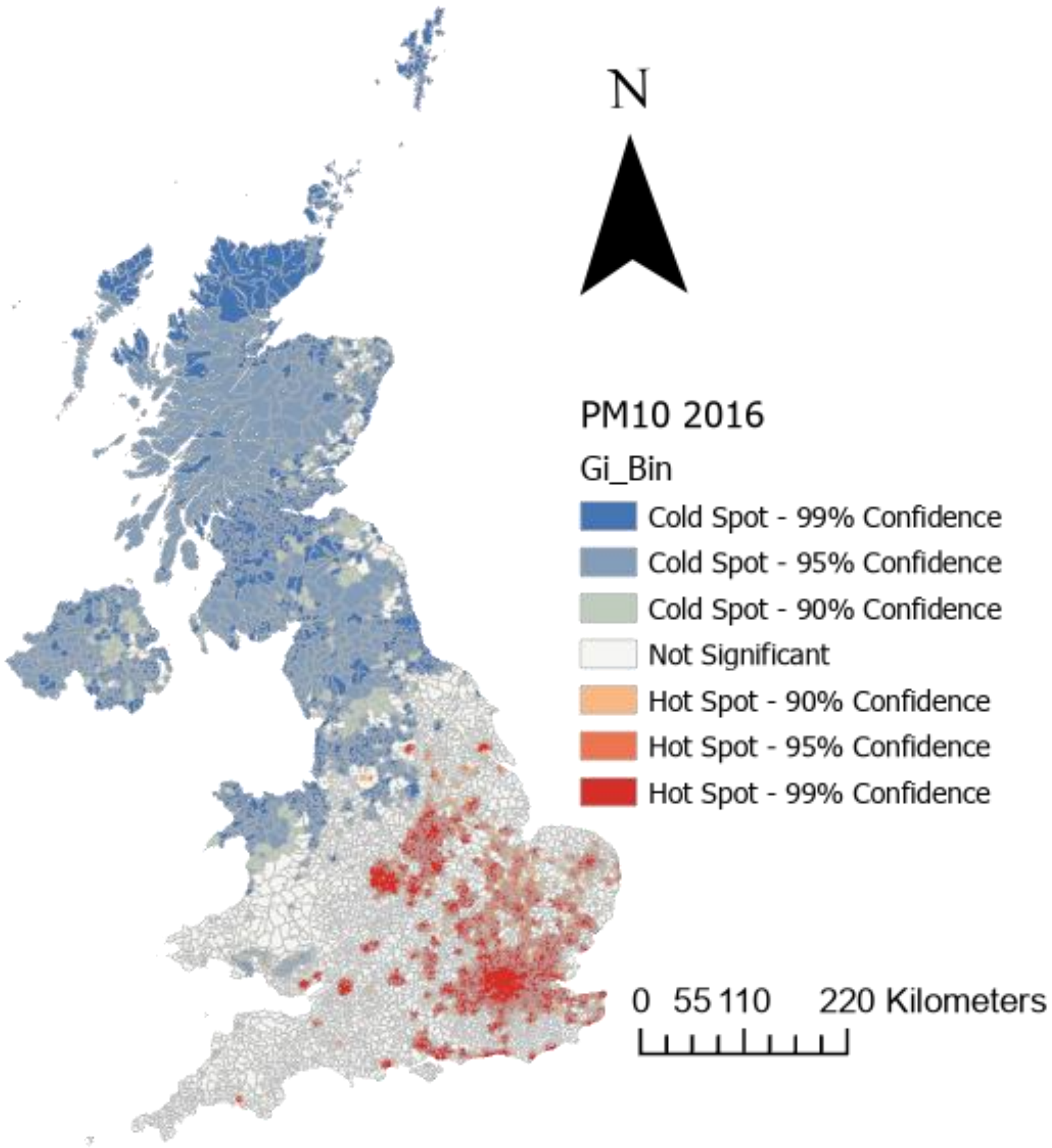

Map 13. PM10 pollutant in 2017

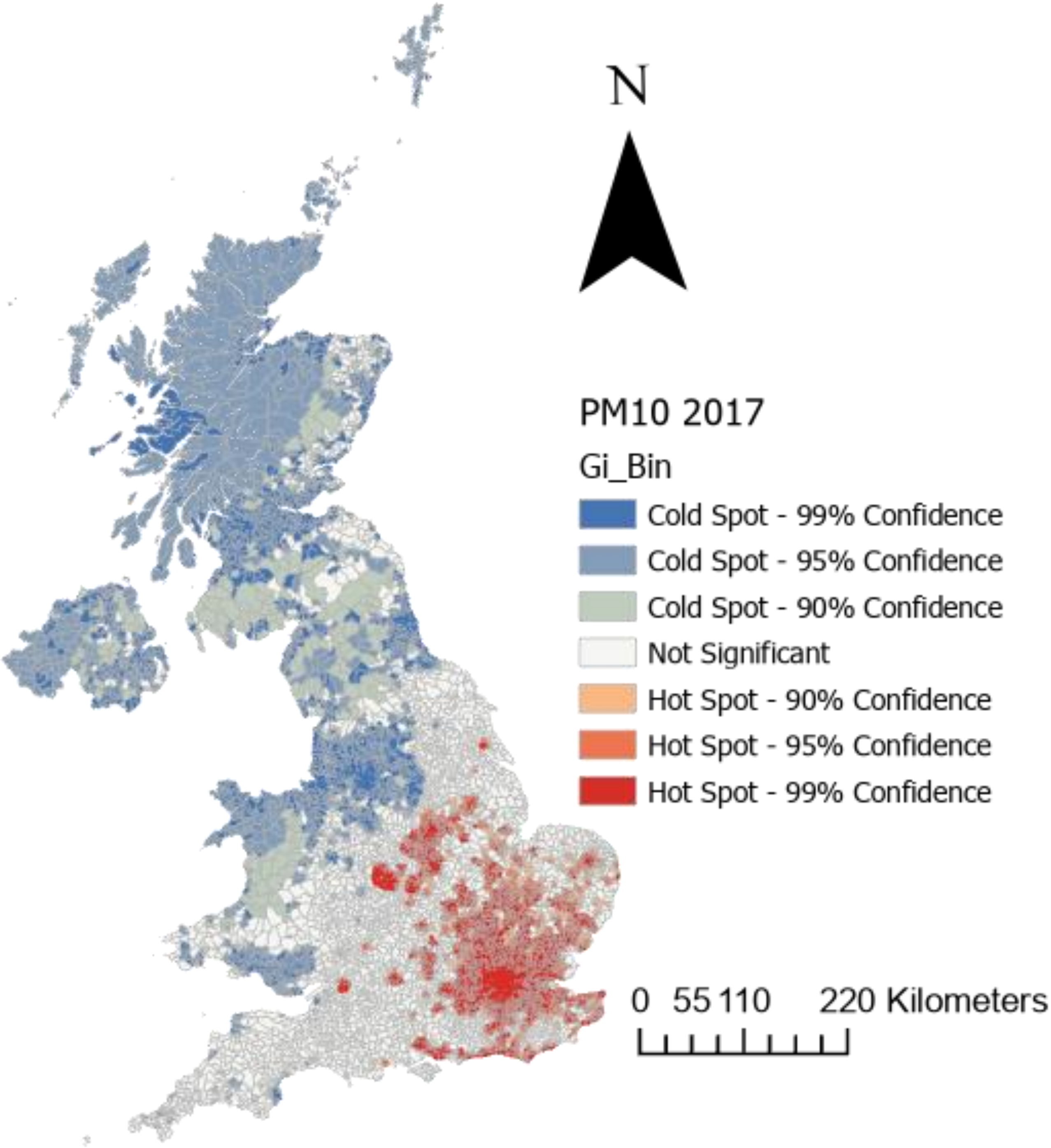

Map 14. PM10 pollutant in 2018

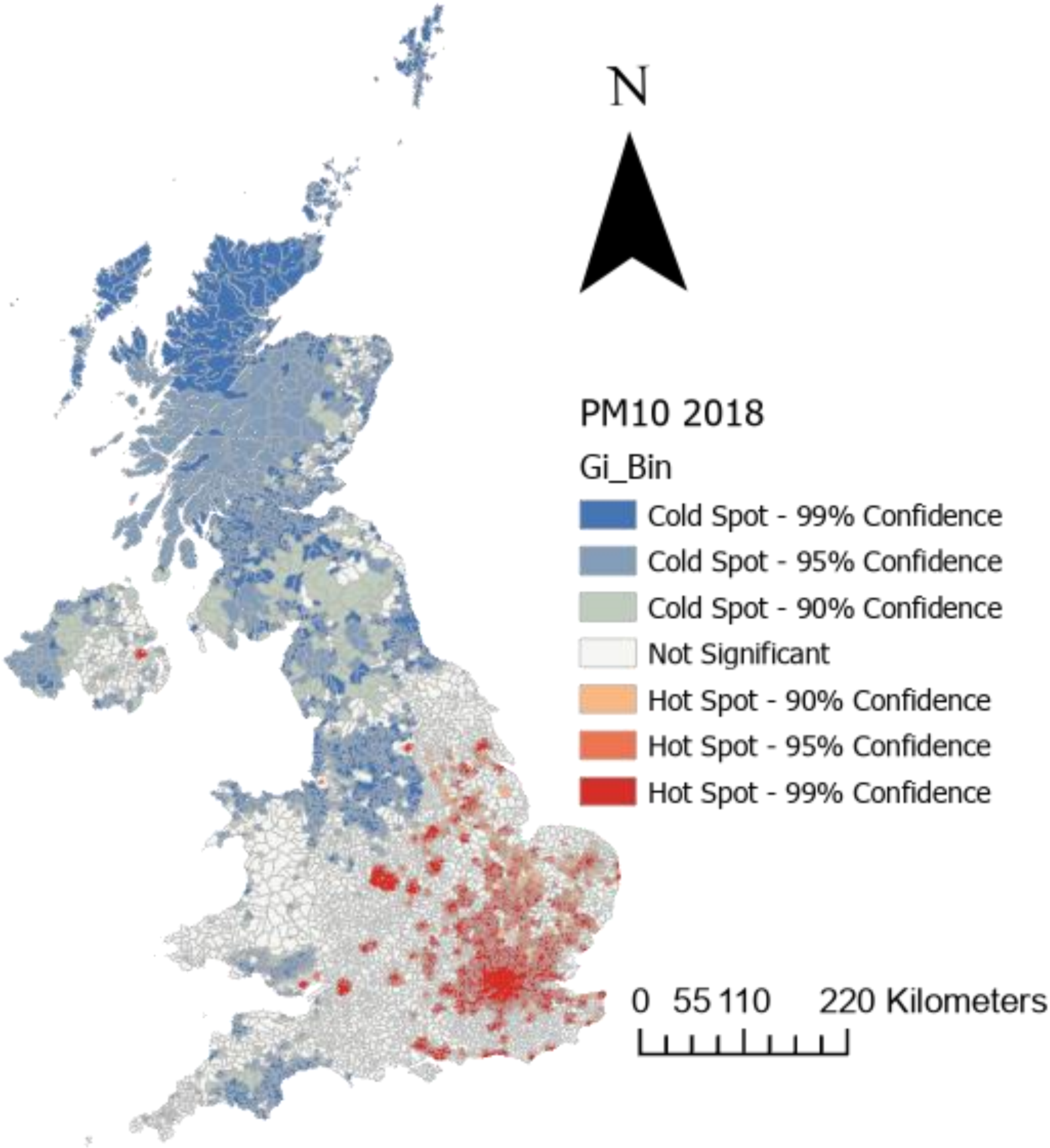

Map 15. PM10 pollutant in 2019

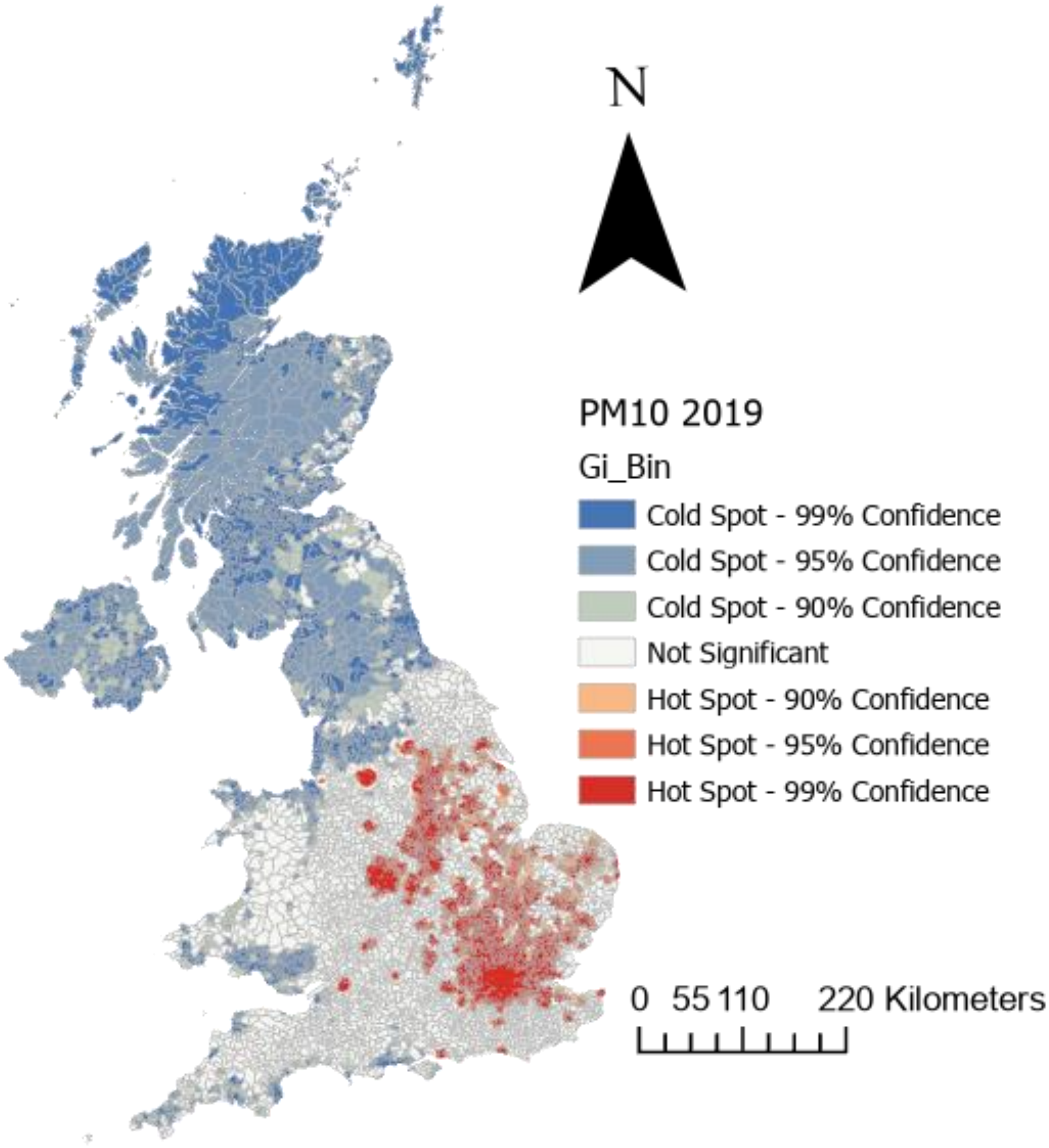

Map 16. PM2.5 pollutant in 2015

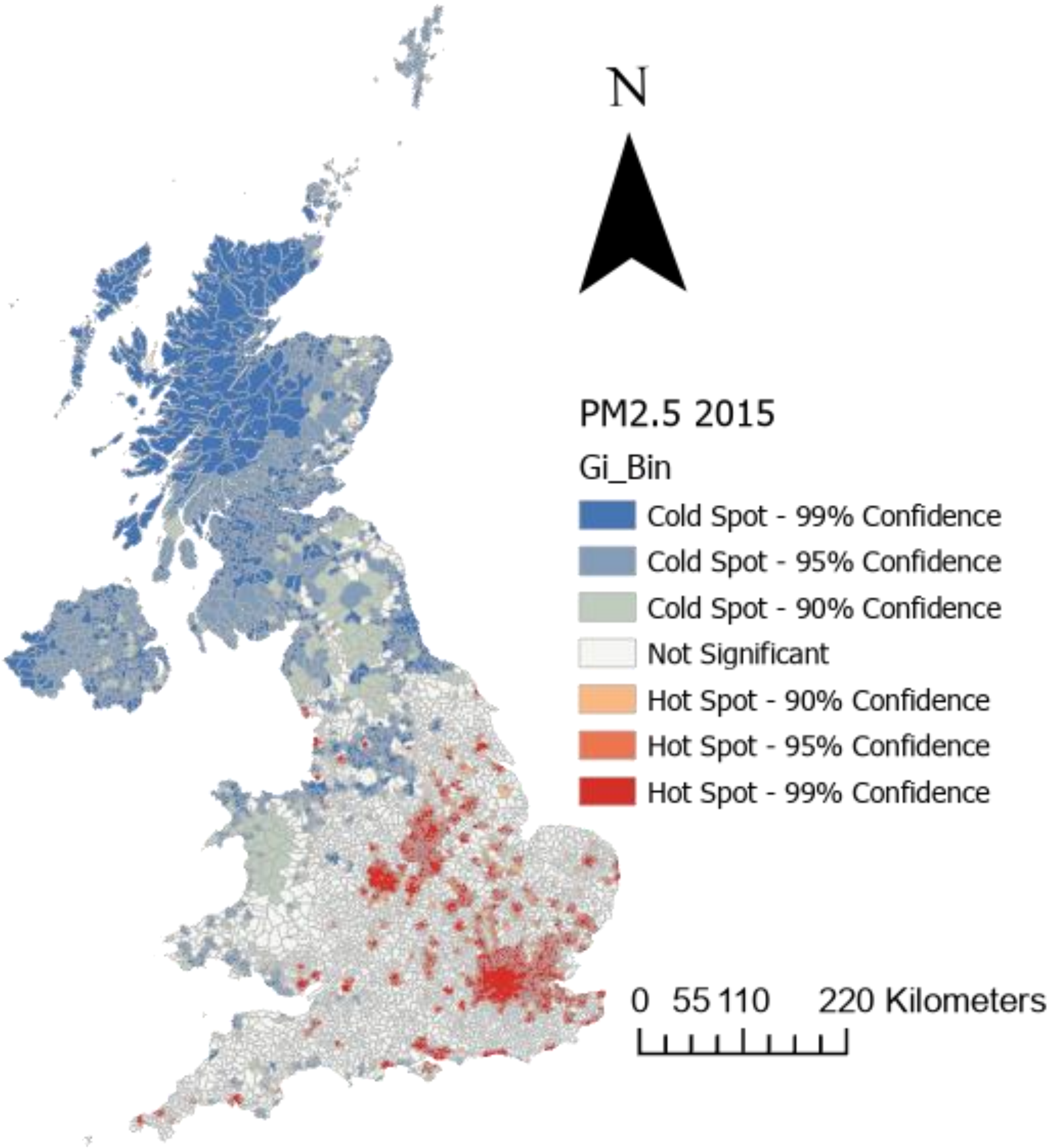

Map 17. PM2.5 pollutant in 2016

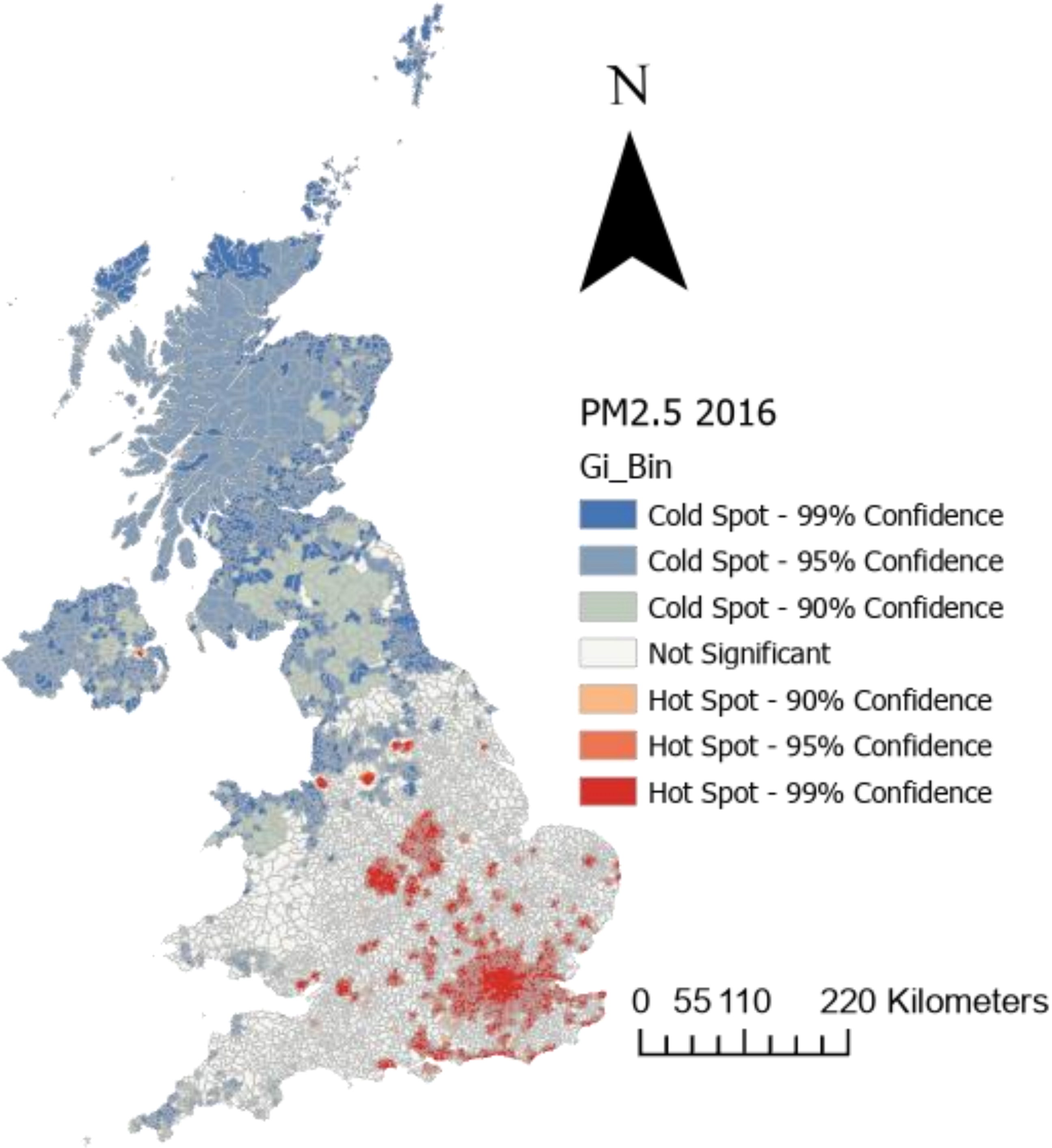

Map 18. PM2.5 pollutant in 2017

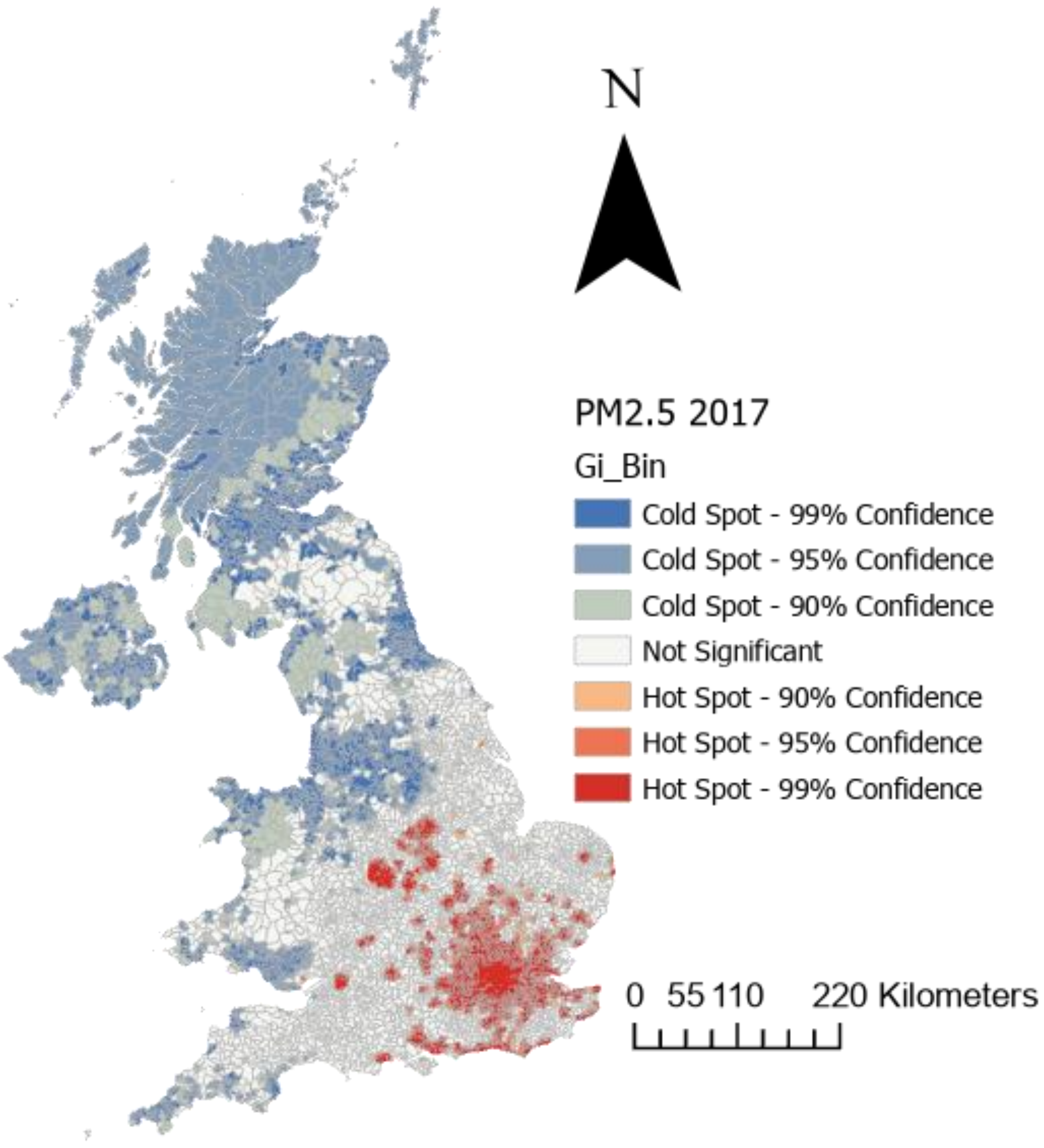

Map 19. PM2.5 pollutant in 2018

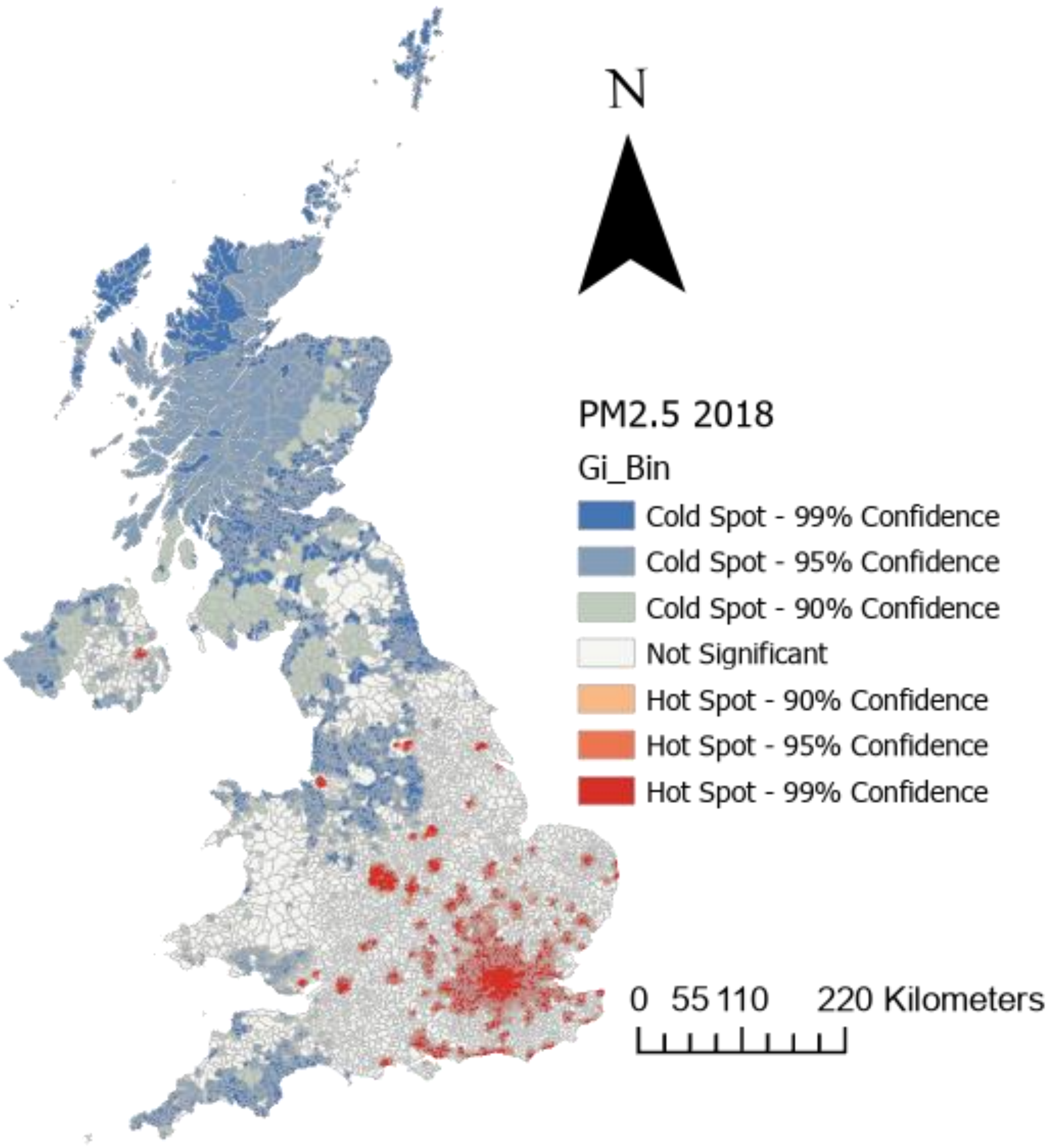

Map 20. PM2.5 pollutant in 2019

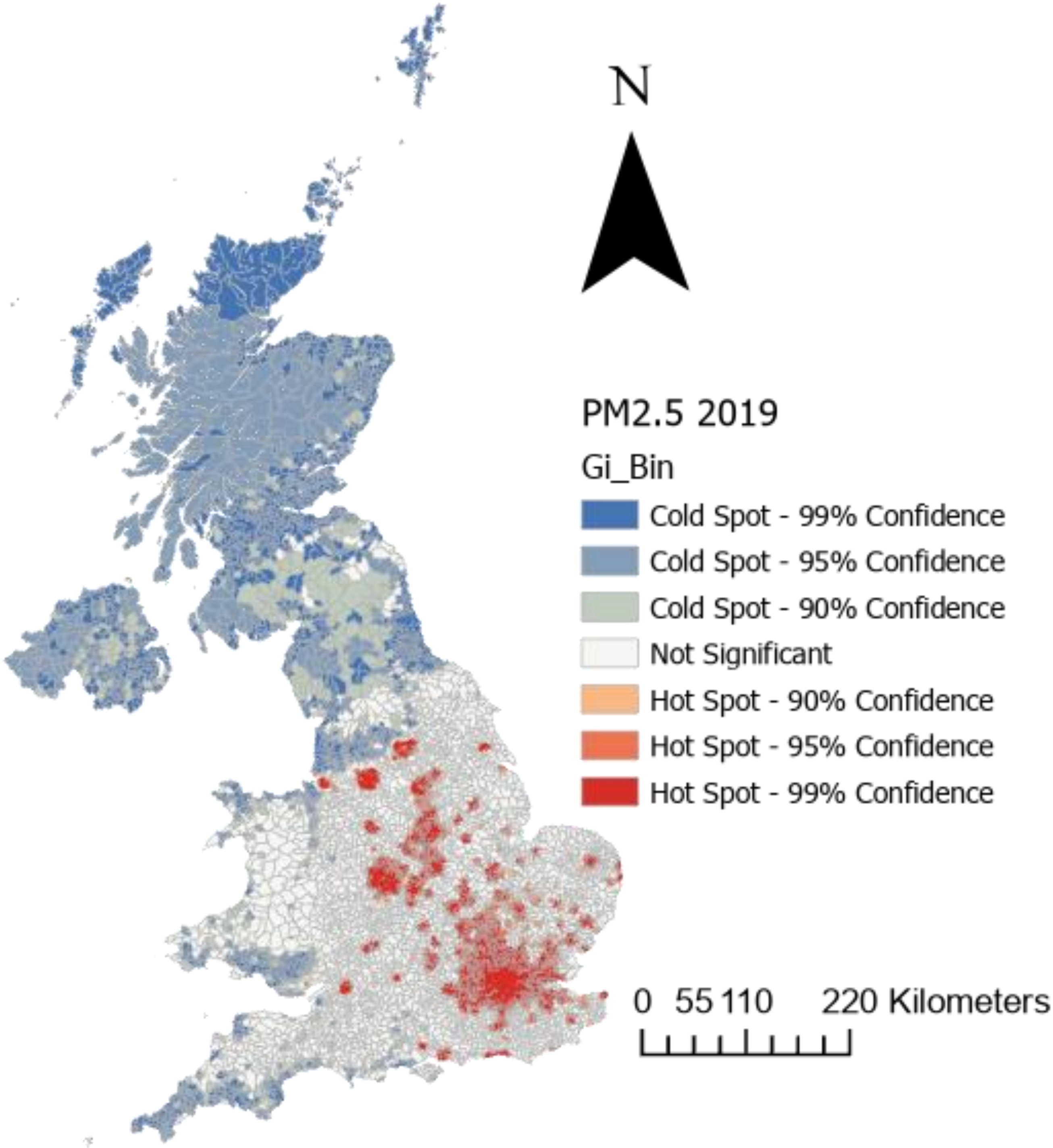

Supplement: S2 File — (PDF) [file pone.0275414.s002.pdf]
